# Mapping organ-associated autoantigenic landscapes in systemic lupus erythematosus

**DOI:** 10.3389/fimmu.2026.1935460

**Published:** 2026-09-18

**Authors:** Ekaterina O. Sosedskaia, Irina A. Ishina, Tatyana M. Reshetnyak, Evgeniy L. Nasonov, Alexander G. Gabibov, Maria Y. Zakharova

**Affiliations:** 1 Shemyakin-Ovchinnikov Institute of Bioorganic Chemistry Russian Academy of Sciences (RAS), Moscow, Russia; 2 Moscow Center for Advanced Studies, Moscow, Russia; 3 V.A. Nasonova Research Institute of Rheumatology, Moscow, Russia; 4 Department of Chemistry, Lomonosov Moscow State University, Moscow, Russia; 5 Faculty of Biology and Biotechnology, National Research University Higher School of Economics, Moscow, Russia

**Keywords:** autoantibodies, autoantigens, B-cell tolerance, epitope spreading, molecular mimicry, organ damage, systemic lupus erythematosus

## Abstract

Systemic lupus erythematosus (SLE) is a systemic autoimmune disease characterized by a remarkably broad and heterogeneous autoantigen repertoire. While epitope spreading, aberrant B-cell activation, and molecular mimicry act in concert with defective apoptotic clearance and sustained type I interferon signaling to drive systemic autoreactivity, many clinical manifestations of the disease are highly localized. The involvement of neuropsychiatric, renal, cutaneous, pulmonary, vascular, and hepatic systems appears to be shaped by tissue-specific autoantigen exposure and local microenvironments, where shared immunopathogenic pathways give rise to organ-associated autoantigenic landscapes. This review develops the concept of organ-associated autoantigenic landscapes, defined here as context-dependent configurations in which autoantibody–autoantigen interactions intersect with regional microenvironments and may contribute to inflammatory cascades, complement activation, and cellular damage. Importantly, organ association does not imply strict organ specificity, as the same circulating autoantibody may acquire distinct clinical relevance across tissues depending on antigen accessibility and local effector conditions. By illustrating how specific autoantibody–autoantigen axes may translate systemic autoimmunity into restricted clinical patterns, this framework provides a unifying model for disease heterogeneity and supports the development of precision, antigen-targeted therapeutic strategies.

## 1 Introduction

Systemic lupus erythematosus (SLE) is a multifaceted autoimmune disease characterized by marked clinical heterogeneity, an unpredictable relapsing-remitting course, and the production of autoantibodies against a wide array of self-antigens (1–3). SLE encompasses heterogeneous clinical phenotypes that differ in the pattern, severity, and temporal evolution of organ involvement. Strikingly, the clinical heterogeneity observed between distinct SLE patient subsets often exceeds the phenotypic variations found between entirely different autoimmune diseases. In recent years, significant efforts have been made to gain a deeper understanding of this disease and how best to treat it (4, 5). A defining immunological feature of SLE is the formation of immune complexes that deposit in tissues and contribute to inflammation and organ damage (6, 7). Among autoimmune diseases, SLE is notable for the extraordinary breadth of its autoantigen repertoire, which includes nuclear, cytoplasmic, membrane-associated, and extracellular targets, most notably double-stranded DNA (dsDNA), histones, small nuclear ribonucleoproteins (snRNPs), and RNA-binding proteins including Ro/SSA and La/SSB (8). Anti-dsDNA antibodies are among the best-characterized SLE-associated autoantibodies and remain important diagnostic and disease-monitoring markers (9–12).

Although SLE is defined by systemic autoimmunity, organ involvement follows non-random and clinically recognizable patterns. The diversity of clinical manifestations and disease courses raises the question of whether SLE represents a single disease or a clinical syndrome. Autoantibodies associated with SLE are not only diagnostic biomarkers of SLE and/or its individual subtypes, but also participate in the development of autoimmune inflammation (13). Distinct autoantibody systems are repeatedly associated with defined clinical phenotypes, supporting a potential mechanistic relationship between autoreactive profiles and regional tissue vulnerability (8, 14). This relationship is illustrated by the associations of anti-dsDNA and anti-C1q antibodies with proliferative lupus nephritis (LN), anti-Ro/La antibodies with photosensitive cutaneous lupus erythematosus (CLE), and antiphospholipid antibodies (aPL) with the vascular thrombosis characteristic of secondary antiphospholipid syndrome (APS) (14–19). However, these serological associations rarely indicate strict organ specificity. The presence of a given autoantibody alone does not fully explain why tissue injury develops in one organ system rather than another in individual patients. This discrepancy suggests that organ-associated pathology in SLE cannot be understood solely by cataloging circulating autoantibody profiles. Instead, a comprehensive understanding requires evaluation of local determinants of pathogenicity, including tissue accessibility, regional inflammatory states, post-translational antigen modifications, and aberrant immune-complex processing (7, 20). Furthermore, vascular injury and the biochemical microenvironment influence whether these autoantigens become accessible to adaptive immune recognition (21).

The mechanisms underlying the extensive diversification of autoreactivity in SLE remain incompletely resolved, but accumulating evidence indicates that a complex network of pathogenic processes acts in concert within genetically predisposed individuals (22, 23). Intra- and intermolecular epitope spreading, aberrant B-cell activation, and molecular mimicry contribute to autoimmune expansion and are further amplified by defective clearance of apoptotic debris, sustained endosomal nucleic acid sensing, and hyperactivated type I interferon signaling (24–28). Together, these processes contribute to defects in central and peripheral B-cell tolerance, increase the availability of nuclear, cytoplasmic, and tissue-associated antigens, enhance dendritic-cell activation and B-cell differentiation, and support class-switched autoantibody production. Within this context, autoreactive B cells may escape tolerance checkpoints, undergo clonal expansion and affinity maturation, and progressively acquire new specificities, thereby expanding the autoantibody repertoire over time (26, 29).

These systemic mechanisms help explain how autoreactivity arises and diversifies, but they do not by themselves explain why particular clinical manifestations localize to specific tissues. The targeted autoantigens can therefore be considered according to how they become immunologically accessible or functionally relevant within local tissue contexts. In SLE, organ-relevant autoantigens may emerge through several distinct but overlapping routes: (i) systemic autoantibody cross-reactivity with peripheral tissues; (ii) exposure of intracellular antigens during apoptosis, inflammation, or tissue injury; (iii) targeting recognition molecules of the classical and lectin complement pathways; and (iv) accessibility of surface, receptor, or extracellular matrix proteins. These routes may coexist within inflamed microenvironments, where ongoing tissue damage can create a feedback loop of antigen release, immune activation, and further diversification of autoreactivity.

In this review, we summarize current evidence on the relationships between specific autoantigens, autoantibodies, clinical manifestations, and mechanisms of SLE pathogenesis. Although SLE is a systemic disease, particular organ manifestations may dominate the clinical phenotype at a given disease stage. To describe autoantibody–autoantigen systems associated with damage to particular organs, we use the concept of organ-associated autoantigenic landscapes. Within this framework, an organ-associated autoantigenic landscape refers to a context-dependent configuration in which an autoantibody–autoantigen interaction acquires local clinical relevance through antigen accessibility, localization, or modification and through tissue-specific inflammatory and effector conditions. This concept describes how systemic immune abnormalities interact with local tissue injury and organ-specific microenvironments, such as those of the central nervous system or kidneys. These landscapes are dynamic and depend on which self-proteins become exposed or modified during cellular stress and how they are recognized by local immune cells, thereby promoting tissue inflammation. Across the organ-focused sections, interpretation of these relationships is guided by the available evidence for clinical association, local antigen accessibility, and tissue-specific conditions that may enable or amplify antibody-mediated effects. Where available, serological associations are considered alongside tissue-based and functional evidence. These considerations are used to structure the interpretation of the available literature rather than to constitute a formal evidence-grading system. This framework allows organ-associated autoantibodies to be interpreted not only as diagnostic or prognostic biomarkers, but also according to their mechanistic status: as putative pathogenic mediators, secondary responses to tissue damage, or unresolved associations requiring further validation.

Literature relevant to this review was identified through targeted searches of PubMed using combinations of terms related to systemic lupus erythematosus, autoantibodies, autoantigens, and the organ manifestations discussed herein. Reference lists of relevant original studies and reviews were also screened to identify additional publications. The selection of autoantibody–autoantigen systems was guided by evidence of association with a defined SLE manifestation, tissue or antigen accessibility within the affected compartment, functional or experimental support for pathogenic relevance, and/or potential utility as an organ-associated biomarker. Where evidence remained limited or inconsistent, the corresponding systems were retained as unresolved associations rather than interpreted as established pathogenic mechanisms.

## 2 Systemic drivers of autoantigen diversification in SLE

Genetic susceptibility provides a permissive background in which defective clearance, endosomal nucleic acid sensing, type I interferon signaling, aberrant B-cell activation, epitope spreading, and molecular mimicry interact to diversify autoreactivity in SLE (22, 23, 26). These mechanisms do not operate in isolation. Rather, they form interconnected pathways linking impaired disposal of apoptotic debris and neutrophil extracellular traps (NETs), sustained innate immune activation, altered B-cell selection, and progressive expansion of autoreactive humoral responses (6, 27, 28, 30). Together, they lower the threshold for the initiation, amplification, and persistence of autoantibody production, thereby contributing to the broad and evolving autoantibody repertoire characteristic of SLE. These systemic drivers are summarized in **Figure 1**, which illustrates how susceptibility genes, defective clearance, nucleic acid sensing, interferon signaling, B-cell activating factor (BAFF)-dependent survival cues, and B-cell activation converge on autoantibody production.

**Figure 1:**
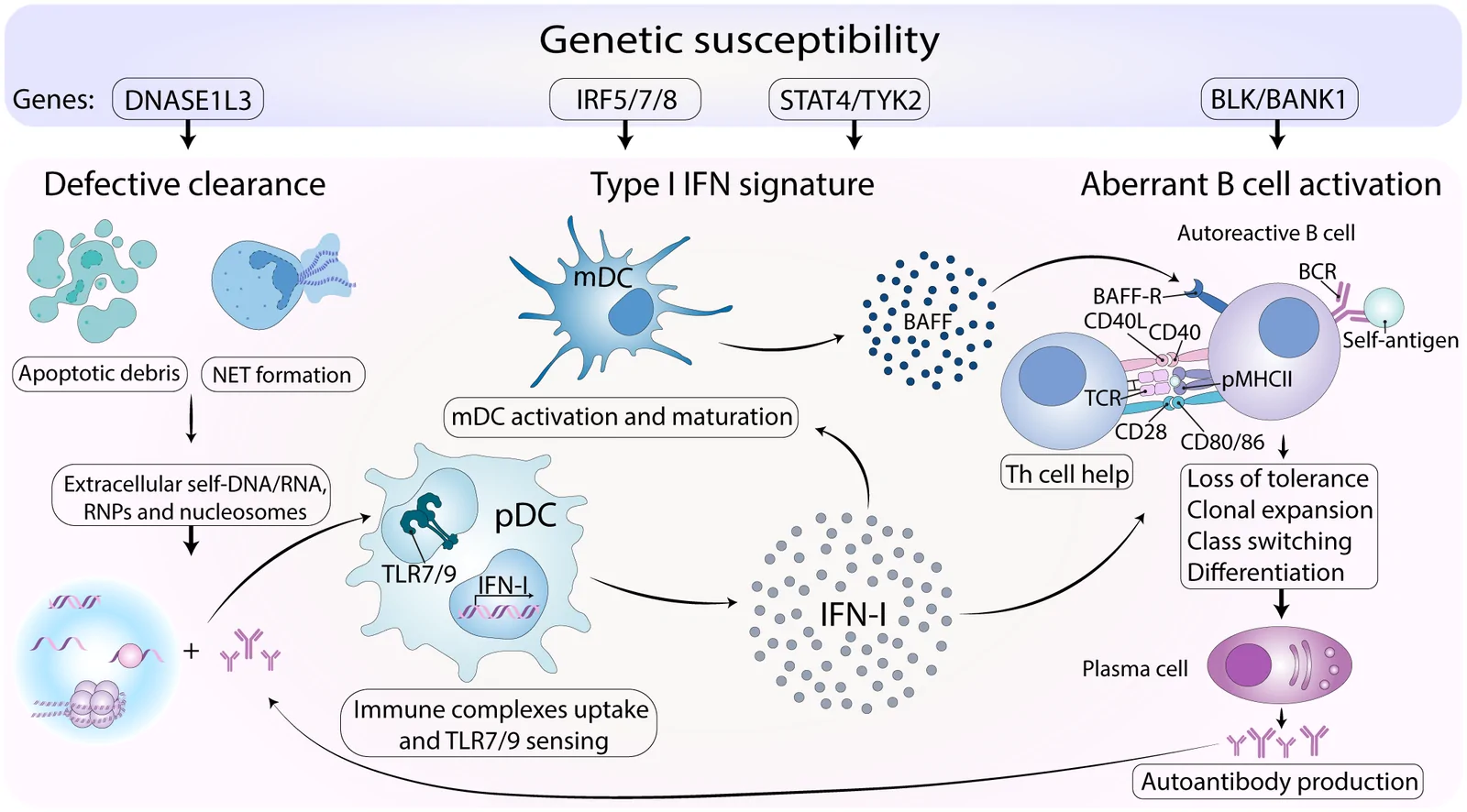
Systemic drivers of autoantigen diversification in systemic lupus erythematosus. The figure summarizes how genetic susceptibility, defective clearance of apoptotic debris and neutrophil extracellular traps, extracellular nucleic acid-containing material, endosomal nucleic acid sensing, type I interferon signaling, BAFF-dependent B-cell survival cues, and aberrant B-cell activation may converge to promote loss of B-cell tolerance and sustained autoantibody production. DNASE1L3-related clearance defects may favor persistence of extracellular DNA/RNA, ribonucleoproteins, and nucleosomes, whereas IRF5/IRF7/IRF8, STAT4/TYK2, and BLK/BANK1 link inherited susceptibility to interferon signaling and altered B-cell activation thresholds. BAFF, B-cell activating factor; BAFF-R, BAFF receptor; BCR, B-cell receptor; CD40L, CD40 ligand; IFN-I, type I interferon; mDC, myeloid dendritic cell; pMHCII, peptide–MHC class II complex; NETs, neutrophil extracellular traps; pDC, plasmacytoid dendritic cell; TCR, T-cell receptor; Th cell, helper T cell; TLR7/9, Toll-like receptors 7 and 9.

### 2.1 Defective clearance, NETs, and nucleic acid sensing

A major systemic driver of autoantigen diversification in SLE is impaired clearance of apoptotic material, immune complexes, and NETs (6, 30). Under physiological conditions, apoptotic cells and nucleic acid-containing debris are efficiently removed or degraded, limiting immune exposure to intracellular self-antigens. In SLE, defective clearance may allow nuclear and cytoplasmic material derived from apoptotic cells and NETs to persist in extracellular compartments, where it can function as a source of damage-associated molecular patterns (DAMPs) (30). These materials may be internalized by plasmacytoid dendritic cells, B cells, and other antigen-presenting cells, promoting endosomal nucleic acid sensing, particularly through Toll-like receptor 7 (TLR7) and Toll-like receptor 9 (TLR9) pathways (28). This process can support type I interferon production, B-cell activation, and autoantibody formation. Recent human genetic evidence has strengthened the causal relevance of this pathway: gain-of-function variants in TLR7 and its endosomal chaperone UNC93B1 can cause monogenic childhood-onset SLE by enhancing responses to endogenous RNA. TLR7 activation therefore provides a mechanistic link between RNA-containing autoantigens, type I interferon production, and the expansion of autoreactive B-cell populations (31, 32).

NETs are a particularly relevant source of extracellular chromatin in SLE. When NET-derived DNA, histones, and associated antimicrobial or nuclear proteins are not efficiently degraded, they may sustain endosomal nucleic acid sensing, immune-complex formation, and inflammatory signaling (33–35). Circulating NET-associated complexes, particularly myeloperoxidase (MPO)–DNA complexes, have been associated with overall disease activity and LN and may therefore represent exploratory biomarkers of active systemic and renal disease, although their clinical utility requires further validation (34). This creates a self-reinforcing loop in which impaired clearance promotes type I interferon production, whereas interferon-driven inflammation further enhances immune-cell activation and lowers the threshold for autoreactive B-cell responses.

DNASE1L3 provides an important example of how defective degradation of extracellular chromatin may promote lupus autoimmunity. DNASE1L3 cleaves DNA–histone complexes present in chromatin, NETs, and DNA-containing microparticles (36). Functional deficiency or reduced extracellular availability of this enzyme has been associated with anti-DNA autoantibody generation and disease progression in patients with SLE and in animal models. When DNASE1L3 function is impaired, undegraded self-DNA can accumulate and stimulate nucleic acid-sensing pathways, thereby amplifying type I interferon production and promoting antibodies against DNA and other antigens released from dying cells.

Genetic evidence further supports the relevance of DNASE1L3-dependent clearance in SLE. Loss-of-function variants in DNASE1L3 can cause severe early-onset SLE, frequently manifesting within the first three years of life and characterized by LN (37, 38). In addition, the common missense variant rs35677470, encoding p.Arg206Cys, has been linked to increased SLE susceptibility. Although earlier studies suggested that this variant produces an inactive form of DNASE1L3 (39), more recent data indicate that the 206Cys variant primarily impairs DNASE1L3 secretion while preserving enzymatic activity. Thus, altered extracellular availability of DNASE1L3 may be sufficient to compromise chromatin clearance and promote autoreactive responses in susceptible individuals (40).

In addition to genetic defects, functional extracellular DNASE1L3 activity may be reduced by neutralizing anti-DNASE1L3 autoantibodies. These antibodies have been reported in subsets of patients with SLE, particularly in nephritis-enriched cohorts, and may represent an acquired mechanism of impaired chromatin degradation (41). Together, genetic and acquired impairments of DNASE1L3 activity provide a direct mechanistic link between inefficient clearance of nuclear debris, sustained nucleic acid sensing, and the emergence of diversified autoantibody specificities.

### 2.2 Type I interferon signaling and B-cell tolerance failure

Localized tissue damage releases novel antigens and additional epitopes from damaged cells, immune complexes, and antigenic structures, thereby perpetuating a self-amplifying cycle of epitope spreading, B-cell activation, and further tissue injury (42). This loop is strongly propagated by type I interferon (IFN-I) signaling, a central feature of SLE pathogenesis.

Under physiological conditions, type I interferon responses are induced during antiviral defense and decline after pathogen clearance. In SLE, nucleic acid-containing immune complexes and persistent innate immune activation can sustain type I interferon signaling beyond its normal antiviral context. This chronic pathway promotes dendritic-cell activation, heightened antigen presentation, B-cell differentiation, immunoglobulin class switching, and survival of autoreactive plasmablasts and plasma cells (27). Elevated circulating type I interferon activity has been associated with active clinical disease phenotypes and with classic SLE autoantibody profiles, including anti-Ro, anti-Sm, anti-ribonucleoprotein, and anti-dsDNA reactivities (43).

Polymorphisms in interferon regulatory factor (IRF) genes, particularly *IRF5* and *IRF7*, as well as variants at the *IRF8* locus, have been associated with SLE susceptibility and help explain the interferon signature observed in many patients with SLE (22). *IRF5* plays a critical role in regulating IFN-I-responsive inflammatory genes and is among the strongest and most consistently replicated SLE susceptibility loci outside the major histocompatibility complex (MHC) region. For example, the *IRF5* rs2004640 risk allele affects alternative splicing and isoform expression, thereby favoring enhanced inflammatory transcriptional responses (23, 44). *IRF7*, a key transcription factor downstream of nucleic acid sensing, has also been linked to SLE susceptibility, and functional variation in *IRF7* can increase transcriptional activity and support enhanced IFN-I-related signaling (45). In addition, *IRF8* has been identified as an SLE susceptibility locus in large-scale multiracial replication studies and has been functionally linked to dysregulated myeloid and antigen-presenting-cell programs (46, 47). These genetic associations suggest that infections or tissue injury that would normally trigger a self-limited antiviral response may instead sustain IFN-I-driven inflammation in genetically predisposed individuals.

Additional susceptibility loci connect cytokine signaling and B-cell activation thresholds to the failure of humoral tolerance (22, 27). Variants in *STAT4* and *TYK2* support the contribution of cytokine-driven JAK–STAT signaling to SLE pathogenesis, linking IL-12/IL-23 and type I interferon signaling pathways to inflammatory T-cell help, dendritic-cell activation, and B-cell differentiation (48, 49). In parallel, B-cell-associated loci such as *BLK* and *BANK1* provide a more direct connection to altered B-cell receptor (BCR) signaling (45, 50). *BLK* encodes a Src-family tyrosine kinase involved in proximal BCR signal transduction, whereas *BANK1* encodes a B-cell scaffold protein that regulates B-cell receptor-induced calcium mobilization and downstream signaling (45, 50). Disease-associated variation in these pathways may tune B-cell activation thresholds, allowing autoreactive clones to receive survival, activation, and differentiation signals more efficiently (26, 51).

These genetic and inflammatory pathways converge on B-cell tolerance failure. In healthy individuals, central and peripheral tolerance checkpoints limit the entry and persistence of autoreactive B cells in the mature repertoire. In SLE, these checkpoints are defective in at least a subset of patients, allowing autoreactive B cells to survive, undergo clonal expansion, and participate in germinal-center or extrafollicular responses (26, 29). BAFF may further support this process by enhancing survival signals for transitional and mature B cells, including autoreactive clones that would otherwise be eliminated (51). Thus, type I interferon, cytokine signaling, BAFF-dependent survival, and altered B-cell receptor signaling cooperate to sustain class-switched autoantibody production and broaden the autoreactive repertoire over time. Similar antigen-driven B-cell responses may occur within ectopic lymphoid aggregates or tertiary lymphoid structures in inflamed tissues, providing local sites for B-cell selection, affinity maturation, and epitope spreading, particularly in organs such as the kidney and skin (52, 53).

### 2.3 Molecular mimicry and epitope spreading

Molecular mimicry and epitope spreading represent complementary mechanisms of autoantibody diversification in SLE (**Figure 2**). Molecular mimicry may breach immune tolerance when exogenous antigens, particularly viral or bacterial components, share sequence, structural, or physicochemical similarities with self-antigens, thereby eliciting cross-reactive immune responses (**Figure 2A**) (54–56).

**Figure 2:**
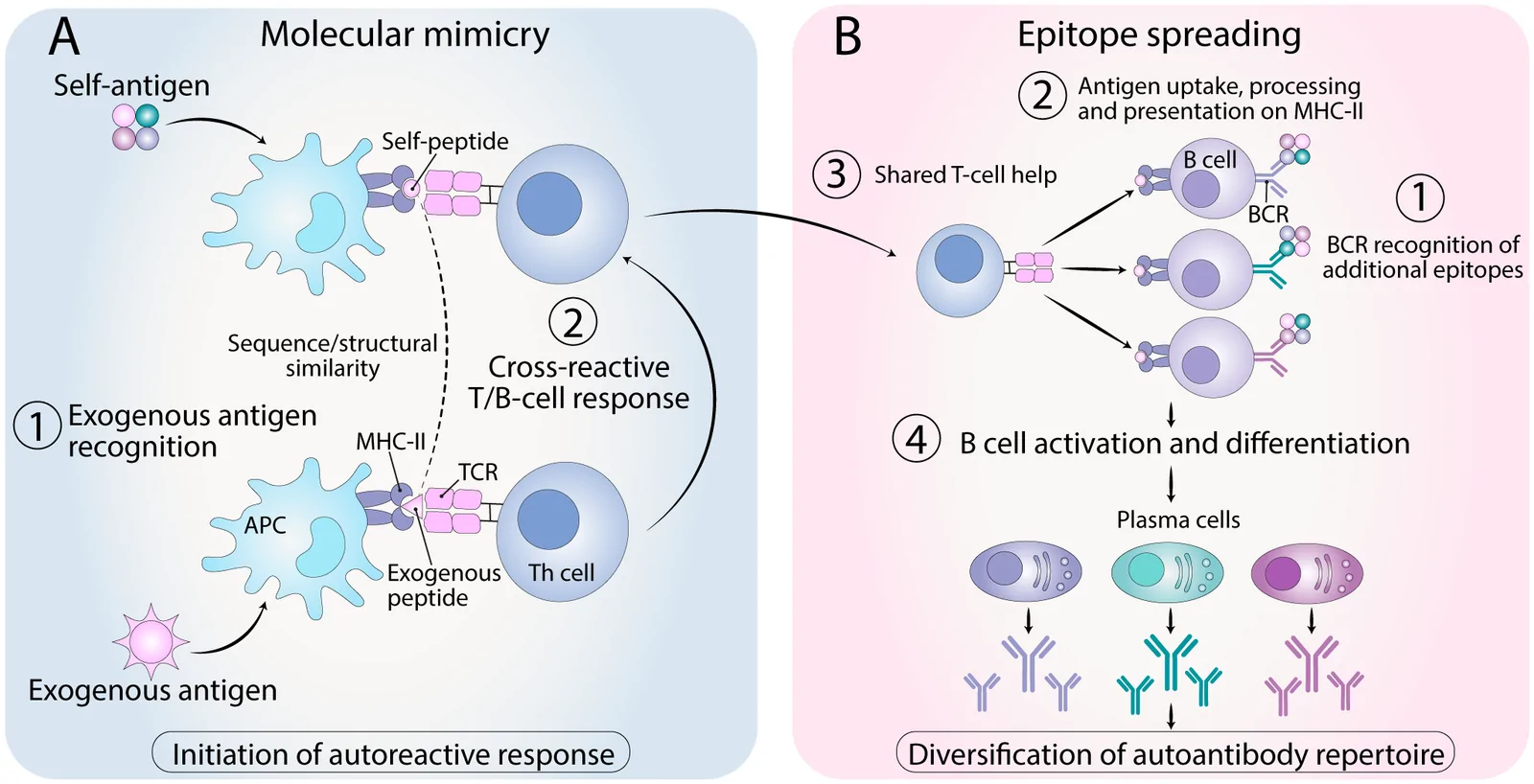
Molecular mimicry and epitope spreading as complementary mechanisms of autoantibody diversification in systemic lupus erythematosus. **(A)** Molecular mimicry may initiate cross-reactive immune responses when exogenous antigens share sequence, structural, or physicochemical similarity with self-antigens, allowing immune recognition of both microbial and self-derived epitopes. **(B)** Epitope spreading may subsequently broaden autoreactivity when antigen uptake, processing, and presentation on MHC class II molecules provide shared T-cell help to B cells recognizing additional epitopes within the same antigen or physically associated antigenic complexes, promoting B-cell activation, plasma-cell differentiation, and diversification of the autoantibody repertoire. APC, antigen-presenting cell; BCR, B-cell receptor; MHCII, major histocompatibility complex class II; TCR, T-cell receptor; Th cell, helper T cell.

Epstein–Barr virus nuclear antigen 1 (EBNA-1) provides one of the best-characterized examples in lupus humoral autoimmunity. An early Ro60 epitope recognized in some patients with SLE cross-reacts with an EBNA-1 peptide, and experimental immunization with either the Ro60 epitope or the cross-reactive EBNA-1 peptide can induce antibodies to multiple Ro epitopes and spliceosomal autoantigens (25). Notably, EBNA-1-related cross-reactivity with human self-antigens has also been implicated in other autoimmune diseases, including multiple sclerosis (57) and primary Sjögren’s syndrome (58), highlighting the capacity of this viral antigen to initiate distinct autoimmune responses in different clinical contexts.

Charge-based mimicry may also contribute to selected anti-dsDNA responses. Anti-DNA antibodies recognize the negatively charged phosphodiester backbone of DNA and may cross-react with other anionic self-antigens (9). Annexin A1 has been proposed as one such candidate because its negatively charged N-terminal region can support charge-dependent interaction with antibodies that also bind dsDNA (59). This mechanism should be interpreted cautiously as a model for selected antibody subsets rather than as a general explanation for anti-dsDNA generation.

Molecular mimicry alone is unlikely to explain the full breadth of autoreactivity in systemic lupus erythematosus. Instead, it may provide an initiating autoreactive specificity that is subsequently amplified by defective clearance, chronic inflammation, type I interferon signaling, aberrant B-cell activation, and epitope spreading.

Epitope spreading is a dynamic process whereby an immune response initially restricted to a primary epitope progressively expands to target additional epitopes within the same antigen or within physically associated antigenic complexes (**Figure 2B**) (60). In SLE, this mechanism provides a plausible explanation for the progressive broadening of autoreactivity from an initial antigenic target to multiple nuclear, cytoplasmic, and tissue-associated molecules. Spreading may occur intramolecularly, when the response extends to additional epitopes within the same autoantigen, or intermolecularly, when B cells recognizing different components of the same ribonucleoprotein, nucleosomal, or spliceosomal complex receive shared T-cell help after antigen uptake, processing, and presentation on MHCII (56). This concept is supported by experimental data showing that immune responses initiated against the proline-rich Sm B/B′ epitopes PPPGMRPP and PPPGIRGP can extend to other Sm B/B′ epitopes and then to additional spliceosomal proteins, including Sm D, U1RNP 70K, U1RNP A, and U1RNP C (24). Longitudinal clinical data showing progressive accumulation of SLE-associated autoantibodies before clinical disease onset further support the concept that lupus autoimmunity can broaden over time (61). Together, these observations suggest that epitope spreading is not simply a theoretical mechanism but a biologically plausible route through which persistent antigen exposure, shared T-cell help, and B-cell activation diversify the SLE autoantibody repertoire over time.

Systemic models of SLE pathogenesis successfully illustrate how broad autoreactivity is sustained, yet they do not fully explain the mechanisms underlying organ-associated vulnerability. Tissue-targeted pathology is ultimately shaped by the local microenvironment, which regulates the accessibility, localization, and biochemical modification of self-antigens. These regional factors may modulate immune-complex trapping, complement activation, vascular injury, barrier disruption, and cellular recruitment.

By applying this localized framework, the next sections analyze key clinical phenotypes of SLE to clarify whether specific autoantibody–autoantigen interactions contribute to tissue injury, reflect secondary tissue damage, or operate through poorly defined pathways (**Table 1**). **Table 1** summarizes this classification by organizing representative autoantibody–autoantigen systems according to their mechanistic route to organ relevance, proposed mechanistic status, highest type of supporting evidence, and disease specificity or overlap with other autoimmune diseases.

**Table 1:** Mechanistic and interpretive classification of organ-associated autoantibody–autoantigen systems in systemic lupus erythematosus.

Mechanistic route to organ relevance	Autoantibody-autoantigen system	Proposed mechanistic status	Highest supporting evidence	Disease specificity/overlap	Key references
Neuropsychiatric SLE					
Cross-reactive receptor target	Anti-dsDNA/anti-NR2-reactive antibodies → GluN2A/GluN2B	Putative pathogenic mediator, CSF-associated candidate biomarker	Meta-analysis of clinical studies; experimental mechanistic evidence	SLE-associated, but not SLE-specific; anti-NR2A/B reactivity also reported in Sjögren’s syndrome	(68–72)
Injury-exposed intracellular neuronal target	Anti-MAP2 antibodies → MAP2	Secondary injury-associated response, candidate biomarker	Clinical case-control evidence	NPSLE-enriched; disease specificity not established	(75, 76)
Intrathecal enrichment	CSF anti-U1RNP antibodies → U1RNP	Unresolved association/candidate CNS biomarker	Small clinical cohort	Shared with mixed connective tissue disease (MCTD)	(64)
Intrathecal/neural-associated autoreactivity	Anti-ribosomal P antibodies → ribosomal P proteins	Unresolved association/candidate stratification biomarker	Clinical association studies	SLE-associated; not NPSLE-specific	(65, 66)
Intrathecal enrichment/neuronal antigen exposure	Anti-TPI antibodies → triosephosphate isomerase	Unresolved association/candidate biomarker	Small clinical studies	Disease specificity not established	(77, 78)
Neural membrane/ganglioside recognition	Anti-ganglioside antibodies → gangliosides	Unresolved association/candidate biomarker	Small pediatric clinical cohort	Not SLE-specific; shared with immune-mediated neurologic diseases, particularly Guillain–Barré and Miller–Fisher syndromes	(79, 80)
Lupus nephritis					
Immune-complex nuclear antigenic substrate, selected cross-reactive nephritogenic subsets	Anti-dsDNA and anti-nucleosome antibodies → dsDNA, nucleosomes, chromatin-containing immune complexes	Putative pathogenic mediator, renal activity biomarker	Prospective longitudinal clinical study; evidence-based/critical reviews	SLE-enriched, but not SLE-exclusive; not kidney-specific	(14, 85–88, 104)
Complement-associated immune-complex target	Anti-C1q antibodies → C1q	Putative pathogenic amplifier, complementary activity biomarker	Systematic review/meta-analysis; longitudinal biopsy-based clinical cohort; experimental mechanistic evidence	Not SLE-specific; shared with other autoimmune and vasculitic diseases	(16, 89–92)
Injury-exposed complement-associated target	Anti-mCRP antibodies → modified/monomeric CRP	Emerging renal activity/prognostic biomarker; pathogenic role incompletely established	Longitudinal biopsy-based clinical cohorts	Not SLE-specific; also reported across other systemic rheumatic diseases	(93–97)
Lectin-pathway pattern-recognition molecules	Anti-ficolin-2 and anti-ficolin-3 antibodies → ficolin-2, ficolin-3	Emerging activity biomarker; mechanistic role unresolved	Biopsy-based clinical cohort evidence	Specificity unresolved; evidence predominantly from SLE/LN cohorts	(98–100)
Cross-reactive glomerular or matrix targets	Cross-reactive anti-DNA antibodies → α-actinin and heparan sulfate	Putative pathogenic mediator, inconsistent circulating biomarker	Experimental mechanistic studies; prospective longitudinal clinical evidence	SLE/LN-enriched association; not established as disease-specific	(102–107)
Planted or locally enriched glomerular antigens	Anti-α-enolase and anti-annexin A1 antibodies → α-enolase and annexin A1	Locally enriched/planted renal autoantigen system, candidate biomarker; pathogenic role unresolved	Human renal-biopsy proteomic evidence	LN-enriched in comparative cohorts; broader disease specificity unresolved	(108, 109)
Stress-induced exposure within inflamed tubulointerstitial tissue	Anti-vimentin antibodies → vimentin	Locally selected tissue-associated response, compartment-specific biomarker; direct pathogenic role unresolved	Human renal-biopsy and *in situ* B-cell clonality evidence	Not established as SLE-specific; LN relevance is compartment-specific	(110–113)
Cutaneous lupus erythematosus					
UV-induced keratinocyte injury, antigen redistribution, and local immune-complex deposition	Anti-Ro/SSA (Ro60 and Ro52/TRIM21)	Putative pathogenic contributor, strong phenotype-associated biomarker	Clinical phenotype-association studies; human *in vitro* and human skin-grafted mouse mechanistic evidence	Shared across CTDs; strongly associated with SCLE but not skin-specific	(17, 20, 25, 115, 118–120, 122, 126, 127)
Apoptosis-associated antigen exposure within the anti-Ro/SSA-associated photosensitive spectrum	Anti-La/SSB	Companion phenotype-associated biomarker, direct pathogenic role unresolved	Clinical association studies	Shared across CTDs; commonly accompanies anti-Ro/SSA; not skin-specific	(20, 115, 123–125)
Apoptosis-associated exposure of intracellular ribosomal antigens	Anti-ribosomal P antibodies → ribosomal P0/RPLP0	Unresolved association/putative contributor	Clinical association studies; experimental murine evidence	Highly SLE-associated; not skin-specific	(20, 116, 129)
UV- or injury-associated redistribution and local inflammatory signaling	Anti-galectin-3 antibodies → galectin-3	Putative pathogenic contributor, candidate activity biomarker	Clinical association study; experimental murine mechanistic evidence	Specificity unresolved; cutaneous association reported in SLE	(116, 130–135)
Direct recognition of exposed basement membrane zone components	Anti-type VII collagen antibodies → type VII collagen	Characteristic autoantibody system in type I BSLE; putative pathogenic mediator	Case-report/review-level clinical evidence; immunopathologic and epitope-mapping evidence	Not SLE-specific; shared with epidermolysis bullosa acquisita and other autoimmune blistering diseases	(117, 136–138)
Vascular abnormalities					
Conformational exposure of phospholipid-binding and coagulation-associated targets	Anti-β2GPI (especially domain I), anticardiolipin, and anti-PS/PT antibodies	Established pathogenic aPL system; criteria aPL are classification markers, whereas anti-PS/PT is a non-criteria risk-associated biomarker	International classification criteria; clinical thrombosis-association studies; experimental mechanistic evidence	Not SLE-specific; shared with primary APS and other aPL-associated disease	(19, 153–156)
Interference with endothelial fibrinolysis or the annexin A5 anticoagulant shield	Anti-annexin A2 and anti-annexin A5 antibodies	Putative pathogenic modifier	Clinical association studies; experimental mechanistic evidence	Not SLE-specific; shared with APS/aPL-associated disease	(157–161)
Recognition of heterogeneous endothelial surface targets	Anti-endothelial cell antibodies (AECA) → heterogeneous endothelial targets	Putative endothelial mediator/candidate vascular biomarker; clinical validation limited	Clinical association studies; *in vitro*/complement-mediated endothelial injury evidence	Not SLE-specific; shared across systemic autoimmune and vasculitic diseases	(146–149)
Recognition of endothelial junctional proteins	Anti-VE-cadherin antibodies → VE-cadherin/CDH5	Candidate endothelial biomarker; pathogenic role in SLE unresolved	Cross-sectional multi-disease clinical study; non-SLE *in vitro* functional evidence	Not SLE-specific; shared across autoimmune diseases	(150)
Functional receptor recognition affecting vascular tone and remodeling	Anti-ETAR antibodies → endothelin receptor type A	Putative agonistic pathogenic mediator; strongest evidence in SLE-associated PAH	Independent clinical cohorts; *in vitro* and *in vivo* experimental evidence	Disease specificity unresolved; strongest evidence in SLE-associated PAH	(151)
NET-associated enzyme exposure and endothelial injury amplification	Anti-MMP-9 antibodies and MMP-9-containing immune complexes → MMP-9	Experimental pathogenic amplifier	Human ex vivo/*in vitro* mechanistic evidence	Disease specificity not established	(152)
Altered recognition of oxidation-associated and lipid-related epitopes	Natural IgM to phosphorylcholine; antibodies to apoB-100 peptides, oxidized LDL, and LPL	Potentially protective humoral responses/unresolved vascular-risk associations	Human observational studies; experimental murine evidence	Not SLE-specific; shared with broader atherosclerosis-related humoral responses	(139–145)
Pulmonary involvement					
Alveolar-capillary and microvascular injury with prothrombotic or proinflammatory effects	Antiphospholipid antibodies → phospholipid-binding proteins	Putative pathogenic modifier, DAH risk association	Systematic review/meta-analysis of clinical studies	Not SLE-specific; shared with APS/aPL-associated disease	(162, 172)
Receptor-directed vascular autoimmunity and pulmonary vascular remodeling	Anti-ETAR antibodies → ETAR	Putative pathogenic mediator in SLE-associated PAH	Clinical cohort studies; *in vitro* and *in vivo* experimental evidence	Disease specificity unresolved; strongest evidence in SLE-associated PAH	(151)
Possible epithelial-immune dysregulation and susceptibility to chronic interstitial injury	Anti-Ro52 antibodies → Ro52/TRIM21	Unresolved association/autoimmune ILD susceptibility biomarker	Systematic review/meta-analysis across autoimmune diseases	Not SLE-specific; shared across CTDs/autoimmune ILD	(173, 174)
Hepatic involvement					
Hepatocyte-associated or injury-exposed target	Anti-ribosomal P antibodies → ribosomal P proteins/hepatocyte membrane-associated P-related target	Suggestive lupus-hepatitis biomarker; putative pathogenic contributor	Small case-control clinical study; *in vitro* mechanistic evidence	SLE-associated, but not liver-specific; cross-disease specificity not established in hepatic studies	(129, 176, 179–181)
Recognition of hepatic membrane-associated targets	Liver membrane autoantibodies (LMAs) → incompletely defined hepatic membrane targets	Historical unresolved association	Historical clinical association study	Disease specificity not established; historical SLE association	(182)

Representative systems are organized according to their mechanisms of organ relevance, proposed mechanistic status, highest type of supporting evidence, and disease specificity or overlap with other autoimmune diseases. The evidence categories indicate the highest type of support represented among the cited studies and are intended as a descriptive evidence map rather than a formal evidence-grading system. Categories may overlap, and organ association does not imply strict organ or disease specificity.

## 3 Manifestations of systemic lupus erythematosus

### 3.1 Neuropsychiatric SLE

Neuropsychiatric systemic lupus erythematosus (NPSLE) encompasses manifestations reported in up to approximately half of patients with SLE. These manifestations present substantial diagnostic challenges because NPSLE is heterogeneous and can reflect inflammatory, antibody-mediated, vascular, thrombotic, metabolic, infectious, medication-related, or unrelated neuropsychiatric processes (62). The pathogenesis of NPSLE is multifactorial, and blood–brain barrier (BBB) dysfunction may facilitate the access of peripheral cytokines and pathogenic autoantibodies to the central nervous system (CNS). Within the CNS, certain antinuclear antibodies (ANAs) may recognize neural components through molecular mimicry or shared epitope specificity. The interpretation of these binding profiles, however, is strongly influenced by tissue preparation and underlying antigen specificity, making it essential to distinguish between non-specific structural binding and true, antigen-specific pathogenic interactions (63).

Several key autoantibody specificities have been associated with the immunological landscape of NPSLE, including anti-NR2-reactive antibodies, cerebrospinal fluid (CSF) anti-U1RNP antibodies, and anti-ribosomal P antibodies. However, their clinical relevance is far from uniform; their diagnostic sensitivity, specificity, and direct pathogenic contributions vary substantially between studies (64–66). Beyond classic lupus markers, active NPSLE has also been associated with higher odds of anti-neuronal antibodies typically used in the diagnosis of paraneoplastic neurological syndromes, including anti-GAD65, anti-CV2, anti-SOX1, and anti-amphiphysin antibodies. While their direct pathogenic contribution remains a subject of ongoing investigation, their strong correlation with active disease may reflect marked polyclonality within B-cell response during lupus flares (67).

Within the neuro-reactive humoral repertoire, anti-N-methyl-D-aspartate receptor (NMDAR) antibodies are the most extensively characterized. The NMDAR functions as a heterotetrameric ion channel composed of two GluN1 and two GluN2 subunits, including GluN2A and GluN2B, and regulates synaptic plasticity at the neuronal membrane. In SLE, the most relevant NMDAR-associated antibodies are anti-dsDNA antibodies that cross-react with NR2 (68). This subset of anti-DNA antibodies may gain access to the CNS when barrier integrity is compromised and can recognize extracellular regions of the GluN2A and GluN2B subunits through molecular mimicry (69). Consequently, antibody binding may induce neuronal dysfunction or neuronal death via excessive NMDAR activation and pathological calcium influx. Given that NMDARs are expressed in hippocampal and amygdala circuits, regions critical for cognition and mood regulation, this mechanism provides a biologically plausible link between anti-NR2-reactive antibodies and selected neuropsychiatric manifestations.

An updated meta-analysis reported that anti-NR2 antibodies were present in the serum or plasma of 24.6% of patients with SLE and 7.6% of healthy donors (70). However, differentiation between NPSLE and SLE without neuropsychiatric manifestations appears more informative when anti-NR2 antibodies are assessed in CSF than in serum or plasma (71). This distinction is important because serum positivity may reflect systemic autoreactivity, whereas CSF positivity may better capture antibody access to the CNS, local immune activation, or BBB dysfunction. Recent experimental work further suggests that lupus autoantibody-mediated neuronal injury may initiate self-sustaining neuroinflammatory loops through high-mobility group box 1 (HMGB1) signaling, providing a potential explanation for persistent cognitive impairment even when systemic disease activity is controlled (72).

It is important to distinguish NPSLE from anti-NMDAR encephalitis, an autoimmune disorder characterized by acute psychiatric symptoms, seizures, movement disorders, and cognitive impairment (73). While both conditions present with overlapping neuropsychiatric symptoms, they differ immunologically: antibodies in anti-NMDAR encephalitis primarily target the GluN1 subunit and can induce receptor clustering, internalization, and synaptic dysfunction (73, 74), whereas in NPSLE, anti-DNA/NMDAR cross-reactive antibodies typically recognize epitopes within the GluN2A/GluN2B subunits, including the consensus pentapeptide motif D/E-W-D/E-Y-S/G (commonly referred to as the DWEYS motif) within the extracellular N-terminal region of GluN2 (69). Thus, similar neuropsychiatric symptoms do not imply identical antigen specificity or pathogenic mechanisms.

Beyond the anti-NR2 axis, other neuro-reactive specificities have been identified, though their direct pathogenic contribution remains less established. Among these, anti-microtubule-associated protein 2 (anti-MAP2) antibodies target an intracellular cytoskeletal protein almost exclusively expressed in neurons that stabilizes microtubules and shapes dendrites (75). Elevated MAP2 levels in CSF are markers of neuronal injury, and patients with NPSLE show elevated levels of IgG antibodies to MAP2, which are associated with diffuse symptoms such as cognitive dysfunction and likely arise secondary to neuronal damage and exposure of intracellular antigens (a “bystander effect” of inflammation) (76). Given its intracellular localization, anti-MAP2 reactivity likely reflects a secondary autoimmune response triggered by structural neuroaxonal injury and may serve as a marker of dendritic injury in NPSLE.

Humoral reactivity in NPSLE also extends to triosephosphate isomerase (TPI), a ubiquitous glycolytic enzyme found in erythrocytes and neurons. Anti-TPI antibodies have been identified in both the peripheral circulation and the CSF of NPSLE cohorts (77, 78), but their functional significance remains uncertain. They may contribute to neuronal metabolic dysfunction or microvascular injury, but may also represent secondary immune responses to tissue stress or damage. The presence of anti-TPI antibodies in both peripheral blood and CSF may reflect a transition from systemic autoreactivity to a more localized CNS-associated immune response.

Anti-ganglioside antibodies (AGAs), which are well characterized in immune-mediated neuropathies such as Guillain–Barré and Miller–Fisher syndromes (79), have also been reported in NPSLE. In a pediatric NPSLE cohort, AGAs were identified in the CSF without corresponding ANA, suggesting intrathecal production rather than passive transfer across a compromised BBB (80). However, broader validation is needed to define their diagnostic and pathogenic relevance in NPSLE.

Reflecting structural neuronal injury, neurofilaments are neuron-enriched intermediate filaments that can be released into extracellular fluids upon axonal damage. Neurofilament light chain (NfL) levels in CSF or blood provide a biomarker of active neuroaxonal injury in NPSLE. Clinical data indicate that serum NfL levels are elevated in NPSLE cases relative to non-NPSLE controls and may help capture active neuroaxonal damage (81, 82). In a retrospective cohort of 144 patients with SLE, however, age and serum creatinine were also identified as independent contributors to serum NfL levels, illustrating that host characteristics may influence the interpretation of organ-associated biomarkers (81). However, NfL should be distinguished from autoantigen-specific antibody responses: it is primarily an injury biomarker, and whether anti-neurofilament antibodies actively amplify neuroinflammation or mainly reflect secondary responses to axonal damage remains to be clarified.

In summary, the complex immunopathology of NPSLE illustrates several distinct routes through which systemic autoreactivity may acquire central nervous system relevance. Cross-reactive anti-dsDNA/anti-NR2 antibodies have plausible pathogenic effects when they access the CNS. Conversely, responses targeting intracellular neuronal structures, such as MAP2 and potentially neurofilament-related antigens, are more indicative of secondary humoral immunity triggered by ongoing neuronal or axonal injury. Meanwhile, other autoantibody profiles, including anti-TPI antibodies, AGAs, anti-ribosomal P, CSF anti-U1RNP, and selected paraneoplastic-type anti-neuronal antibodies, may help stratify patients into immunological subsets, even though their precise pathogenic contributions remain variable and incompletely understood.

### 3.2 Lupus nephritis

LN remains one of the major organ manifestations of SLE and an important determinant of chronic kidney disease and long-term morbidity. Current recommendations for the management of SLE with kidney involvement continue to emphasize early recognition, histological classification, and longitudinal monitoring of renal activity (83, 84). Among conventional serological markers, anti-dsDNA antibodies correlate with overall disease activity and renal flares and therefore remain central to clinical monitoring (85). However, their specificity for renal involvement is limited, as elevated levels are frequently observed in patients without overt nephritis. Moreover, critical analyses have emphasized the heterogeneity of anti-dsDNA responses, showing that individual isotypes or subclasses do not consistently improve discrimination of renal involvement or reliably track longitudinal renal activity (14, 86).

This limitation has prompted increasing attention to kidney-associated autoantibodies and complement-related mechanisms that may explain renal involvement in LN. In this respect, LN represents one of the clearest organ manifestations in which circulating autoantibodies, glomerular immune deposits, complement activation, and locally enriched renal targets can be considered together (87, 88).

Complement activation is a central effector pathway in immune complex-mediated renal injury. C1q, the recognition subunit of the classical pathway, binds immune complexes and initiates complement activation, while also contributing to the clearance of immune complexes and apoptotic debris (16). In SLE, impaired clearance and immune-complex deposition may render surface-bound C1q antigenic and promote anti-C1q autoimmunity (89). Clinically, anti-C1q antibodies are enriched in active LN, particularly in proliferative forms. In a recent biopsy-based cohort, 47 of 61 patients had proliferative LN, with higher anti-C1q and anti-dsDNA titers and lower C3/C4 levels than patients with non-proliferative LN. In the longitudinal component of the same study, persistent high anti-C1q levels and non-use of hydroxychloroquine independently predicted renal flares, illustrating that serological risk may be modified by treatment exposure (90).

Experimental data suggest that anti-C1q antibodies can deposit in glomeruli together with C1q, but do not induce overt renal injury in the absence of pre-existing C1q-containing immune complexes along the glomerular basement membrane (GBM). Their pathogenic relevance appears more evident when such complexes are already present, whereupon anti-C1q may amplify glomerular inflammation through both complement-dependent mechanisms and Fcγ receptor engagement (91). Meta-analytic data support an association between anti-C1q antibodies and LN, although diagnostic performance is only moderate. In the hierarchical summary receiver operating characteristic (HSROC) meta-analysis, at the median study specificity of 73.5%, the corresponding estimated sensitivity for distinguishing patients with versus without a history of LN was 70.4% (95% CrI, 57.4–81.6%). For active versus inactive LN, the median specificity was 80.0%, with a corresponding estimated sensitivity of 75.7% (95% CrI, 46.8–91.3%). These findings support the use of anti-C1q as a complementary rather than stand-alone biomarker (92).

C-reactive protein (CRP) represents another target within complement-associated immune pathways. Circulating pentameric CRP can dissociate into monomeric or otherwise modified CRP (mCRP) at sites of tissue injury, exposing neoepitopes and enabling C1q binding (93). In LN, the relevant autoantibody response is directed largely against mCRP rather than native circulating pentameric CRP (94). Earlier work showed that anti-CRP levels correlate with histopathological activity, and baseline positivity predicted a poor response to therapy (95). In biopsy-proven LN, anti-CRP antibodies were found predominantly in active disease, correlated with the Systemic Lupus Erythematosus Disease Activity Index (SLEDAI), and predicted a poor 2-year renal outcome (96). More recent work further suggests that combined anti-C1q and anti-mCRP epitope-specific responses may be associated with renal prognosis, supporting the concept of coordinated complement-related autoreactivity in LN (97). These findings support anti-CRP antibodies as potentially informative markers of inflammatory renal injury, although their value as independent pathogenic mediators remains less clearly established.

Beyond the classical complement pathway, autoreactivity in LN extends to lectin pathway pattern-recognition molecules (PRMs). Elevated anti-ficolin-1, anti-ficolin-2, and anti-ficolin-3 autoantibodies have been reported in SLE, whereas anti-mannose-binding lectin (MBL) antibodies in that study were associated with disease activity rather than being significantly increased overall (98). Among these lectin pathway autoantibodies, both anti-ficolin-2 and anti-ficolin-3 antibodies have been associated with active LN. The anti-ficolin-2 and anti-ficolin-3 studies were based on the same underlying cohort of 165 patients with SLE and 48 healthy controls; among 77 patients with active SLE, 36 had biopsy-proven active LN and 41 had active disease without renal involvement (99, 100). Anti-ficolin-2 antibodies, assessed by ELISA using a 95-AU positivity cut-off, were detected in 86% of patients with active LN and were significantly enriched in proliferative disease (99). Anti-ficolin-3 antibodies, assessed by ELISA using a 70-AU positivity cut-off derived from the healthy-control distribution, were detected in 25 of 36 (69%) patients with active LN compared with 13 of 41 (32%) patients with active nonrenal SLE and were enriched in proliferative LN (85% vs 22%) (100). Notably, the combination of anti-ficolin-2, anti-ficolin-3, and anti-C1q demonstrated 98% specificity for active LN in this cohort (99), supporting the possibility of coordinated autoreactivity involving both lectin and classical complement pathway components. In addition, *FCN2* promoter polymorphisms have been associated, in specific populations such as Egyptian pediatric cohorts, with altered ficolin-2 levels and increased susceptibility to LN (101). Collectively, these findings are consistent with coordinated complement-directed autoreactivity that may amplify immune complex-driven glomerular inflammation. However, given the relatively small renal subgroup and study-specific assays, these findings require validation in independent cohorts before these markers can be considered robust clinical biomarkers of LN activity.

Complement dysregulation alone does not fully account for renal tropism. Experimental evidence indicates that subsets of anti-DNA antibodies may acquire affinity for glomerular structures through cross-reactivity. α-Actinin, an actin-binding cytoskeletal protein expressed in podocytes and mesangial cells and important for glomerular structural integrity, was identified as a cross-reactive renal target of pathogenic anti-DNA antibodies (102). Subsequent studies using human lupus monoclonal antibodies supported binding to α-actinin, glomerular deposition, and podocyte injury in experimental models (103). Despite this mechanistic plausibility, the clinical relevance of circulating anti-α-actinin antibodies is less consistent: in prospective biopsy-proven LN cohorts, anti-α-actinin titers have not robustly correlated with proteinuria, serum albumin, or remission status (104). More recently, IgG anti-membrane autoantibodies were shown to be enriched in biopsy-proven LN, with α-actinin identified as a major target antigen and a predominance of the IgG2 subclass (105). Thus, α-actinin is unlikely to be a robust stand-alone biomarker of renal activity, but remains an instructive model of renal-relevant cross-reactivity.

This cross-reactivity paradigm also extends to the GBM. Experimental murine studies showed that anti-dsDNA autoantibodies can bind directly to the GBM, colocalize with heparan sulfate proteoglycans in the GBM and mesangial matrix, activate complement, and promote glomerular injury (106). In patients with LN, elevated anti-heparan sulfate antibody levels have been reported together with functional loss of GBM heparan sulfate proteoglycans, alterations that may contribute to proteinuria and impairment of the filtration barrier (107). Together, α-actinin and heparan sulfate illustrate how anti-DNA reactivity may acquire renal relevance through cross-reactive recognition of glomerular structures, although the strength of clinical association differs between targets.

In contrast to cross-reactive mechanisms, proteomic analyses of laser-microdissected glomeruli identified organ-focused IgG2 autoantibodies against α-enolase and annexin A1 within immune deposits of biopsy-proven LN (108). α-Enolase is a glycolytic enzyme that can also function as a cell-surface plasminogen receptor, whereas annexin A1 is a Ca2+-dependent phospholipid-binding protein involved in membrane dynamics and anti-inflammatory signaling; both can be expressed in glomerular cells. Anti-α-enolase IgG2 did not cross-react with dsDNA and recognized defined epitopes, supporting an organ-focused immune response rather than secondary cross-reactivity. Serum anti-α-enolase IgG2 levels have been correlated with severe proteinuria and impaired renal function, particularly in classes III and V LN (109). This distinction is important because it suggests that at least some renal autoantigens are not merely secondary targets of systemic anti-dsDNA responses, but may represent locally enriched or planted antigens within glomerular immune deposits.

The tubulointerstitium represents a distinct renal compartment of major pathogenic relevance in LN. Severe tubulointerstitial inflammation (TII) is associated with poor renal outcomes and is increasingly recognized as a site of localized, antigen-driven humoral immunity that may operate partly independently of glomerular immune-complex deposition (110). In this context, vimentin has emerged as a dominant target of *in situ* B-cell responses within inflamed tubulointerstitial tissue (111). As an intracellular intermediate filament protein, vimentin is significantly upregulated and becomes antigenically accessible during local cellular stress, inflammation, and tissue injury (112). Its immunological targeting supports the concept that renal autoantigenicity in LN is compartment-specific. Moreover, anti-vimentin clones in TII show evidence of local affinity maturation and selection, consistent with an active tissue-embedded autoimmune response rather than simple deposition of circulating antibodies (113). Thus, anti-vimentin reactivity is best interpreted not as a general biomarker of LN but as a marker of severe tubulointerstitial immune pathology associated with progressive renal damage.

Beyond antigen specificity, antibody subclass and Fc glycosylation may modify complement activation and Fcγ receptor engagement, providing an additional determinant of renal pathogenicity (114).

Collectively, these findings illustrate several overlapping routes through which systemic autoimmunity acquires renal relevance in LN. Anti-dsDNA remains a conventional marker of systemic and renal activity but is not kidney-specific. Anti-C1q, anti-CRP or anti-mCRP, and anti-ficolin antibodies place complement and pattern-recognition molecules at the center of immune-complex amplification. α-Actinin and heparan sulfate exemplify cross-reactive glomerular or matrix-associated targets of anti-DNA antibodies, whereas α-enolase and annexin A1 support the concept of locally enriched or planted glomerular antigens. Vimentin highlights a compartment-specific, locally selected humoral response in tubulointerstitial inflammation. Thus, renal autoantigenicity in SLE should be interpreted as a layered organ-associated landscape shaped by immune-complex deposition, complement activation, glomerular cross-reactivity, planted antigens, compartment-specific inflammation, and local tissue injury rather than by any single nephritis-determining autoantibody.

### 3.3 Cutaneous lupus erythematosus

Cutaneous involvement is among the most frequent organ manifestations of SLE: up to 70–85% of patients develop lupus-specific or lupus-associated skin lesions during the disease course. CLE encompasses a spectrum from skin-limited disease to cutaneous manifestations accompanying systemic SLE and is classically divided into acute CLE (ACLE), subacute CLE (SCLE), chronic CLE (CCLE, including discoid lupus), and rarer forms such as bullous systemic lupus erythematosus (BSLE) (18, 115).

The emergence of cutaneous autoantigens in SLE is strongly influenced by the inflammatory skin environment. Apoptosis of keratinocytes, often triggered by ultraviolet light, generates membrane blebs that cluster autoantigens such as Ro/SSA and ribosomal P proteins, thereby exposing the immune system to a concentrated array of intracellular autoantigens (20). In CLE, molecular mimicry may also contribute to Ro60-centered autoimmunity in the anti-Ro/SSA-associated photosensitive spectrum. Cross-reactivity between an early Ro60 epitope and an EBNA-1 peptide supports a model in which Epstein–Barr virus (EBV)-related mimicry may initiate autoreactivity that is subsequently amplified by epitope spreading (25). In skin, ultraviolet-induced keratinocyte apoptosis may then expose or cluster Ro/SSA antigens, increasing their local pathogenic relevance.

Many systemic autoantibodies typical of SLE, including ANA, anti-dsDNA, and anti-Sm, can be detected in cutaneous disease but lack skin specificity. By contrast, several autoantibody systems appear enriched in CLE or linked to defined cutaneous phenotypes, particularly anti-Ro/SSA, anti-ribosomal P0, anti-galectin-3, and antibodies to type VII collagen or other basement membrane zone components in BSLE (17, 116, 117).

Anti-Ro/SSA antibodies are directed against two structurally and functionally distinct autoantigens that are often grouped clinically within the SSA/Ro system: Ro60 (TROVE2) and Ro52 (TRIM21). Ro60 is an RNA-binding protein associated with Y RNAs and implicated in small-RNA quality control, cellular stress responses, and regulation of endogenous retroelement-derived transcripts (118), whereas Ro52/TRIM21 is a cytosolic E3 ubiquitin ligase that modulates innate immune signaling, including type I interferon pathways (119). Mechanistically, Ro60-centered autoimmunity is linked to interferon-driven lupus through the binding of endogenous Alu RNAs and related inflammatory gene programs (120).

Clinically, anti-Ro/SSA antibodies are strongly associated with SCLE, especially photosensitive, non-scarring annular or papulosquamous eruptions on sun-exposed skin, and they also constitute the principal maternal autoantibody system associated with neonatal lupus (115, 121, 122). High-titer precipitating anti-Ro/SSA responses are much more typical of SCLE than of discoid lupus erythematosus, in which lower-titer reactivity may occur without conferring the same phenotype (17). Anti-La/SSB antibodies may accompany anti-Ro/SSA responses in CLE, particularly within the photosensitive SCLE spectrum, but they are less strongly associated with a distinct cutaneous phenotype than anti-Ro/SSA and are best interpreted as companion rather than primary cutaneous autoantibodies (115, 123). In SCLE, anti-Ro/SSA antibodies are detected in approximately 80% of patients, whereas anti-La/SSB antibodies are present in about 30–40%. Similar serological frequencies have been reported in idiopathic and drug-induced SCLE (115). In a separate SLE cohort, anti-SSB antibodies were found in 25.7% of patients and were associated with higher frequencies of cheek erythema (68.4% vs 38.2%) and alopecia (31.6% vs 9.1%), together with increased anti-Ro positivity (124). Biologically, La/SSB shares apoptosis-associated exposure pathways with Ro/SSA (125). However, its role in CLE is best interpreted in the context of anti-Ro/SSA-associated photosensitive disease rather than as an independent skin-specific autoantibody system.

Experimental data provide biologic plausibility for a contributory role of anti-Ro/SSA in cutaneous injury. Ultraviolet irradiation induces keratinocyte apoptosis and promotes surface translocation or clustering of Ro/SSA antigens, favoring local binding of circulating antibodies and immune-complex deposition at the dermo-epidermal junction (126). In a human skin-grafted mouse model, purified anti-Ro/SSA IgG reproduced a lupus-band-like pattern and induced histologic changes resembling SCLE (127). These observations support a contributory pathogenic role for anti-Ro/SSA in at least a subset of photosensitive CLE, while also underscoring that anti-Ro/SSA is not skin-specific within SLE.

This context dependence is further illustrated by maternal anti-Ro/SSA-associated fetal cardiac injury. Maternal anti-Ro/SSA IgG crosses the placenta, but cardiac injury, including congenital heart block, develops only in a minority of exposed fetuses, indicating that antibody exposure alone is insufficient. Experimental evidence supports a model in which Ro/SSA antigens become accessible on apoptotic fetal cardiocytes, followed by impaired apoptotic-cell clearance and macrophage-mediated inflammatory and profibrotic signaling. Thus, fetal cardiac relevance appears to depend on the combination of transplacental antibody access, developmental tissue susceptibility, apoptosis-associated antigen exposure, and local effector responses rather than on maternal anti-Ro/SSA positivity alone (121, 125, 128).

Ribosomal P proteins (P0, P1, and P2) are acidic phosphoproteins of the 60S ribosomal subunit and well-established SLE-associated antigens. In studies screening SLE sera against epidermal extracts, a 35-kDa cutaneous antigen was identified as acidic ribosomal protein P0 (RPLP0) (116).

As a cytosolic ribosomal protein, RPLP0 is normally shielded from immune recognition. However, keratinocyte apoptosis, a prominent feature of photosensitive CLE, generates membrane blebs enriched in ribosomal components together with other lupus autoantigens such as Ro/SSA (20). This provides a plausible mechanism by which ribosomal P proteins become locally antigenically accessible in inflamed skin. Anti-ribosomal P seropositivity has been associated in some studies with increased disease activity and with cutaneous features such as photosensitivity, alopecia, and cutaneous vasculitis, with contributions from all three ribosomal P components (P0, P1, and P2) (129). Purified anti-RPLP0 antibodies induced lupus-like histologic changes after intracutaneous injection in mice (116). Thus, RPLP0 is better viewed as an intracellular antigen that acquires cutaneous relevance in the context of apoptosis-driven antigen exposure rather than as a constitutive skin-specific structural antigen.

Galectin-3 is a β-galactoside-binding lectin expressed in keratinocytes, fibroblasts, endothelial cells, and various immune cells, where it regulates cell adhesion, apoptosis, inflammatory signaling, and tissue repair (130). In skin, galectin-3 contributes to keratinocyte proliferation, migration, differentiation, and wound healing, and its expression and subcellular distribution are modulated by ultraviolet irradiation and oxidative stress (131, 132). During apoptosis or epithelial injury, galectin-3 may redistribute to the cell surface or extracellular space, where it can influence immune cell recruitment and cytokine production (133, 134). In the same proteomic approach that identified RPLP0, a 25-kDa antigen preferentially recognized by sera from patients with cutaneous lesions was identified as galectin-3 (116). Anti-galectin-3 antibody levels were elevated in patients with active cutaneous disease and declined with clinical improvement. Moreover, purified anti-galectin-3 antibodies also induced lupus-like histopathologic changes and local IL-1β production after intracutaneous injection (135). These findings make galectin-3 a plausible cutaneous target, although broader validation across CLE phenotypes remains limited.

BSLE is a rare blistering manifestation of SLE characterized by tense subepidermal blisters, often arising on sun-exposed skin in the context of established SLE, although it may occasionally be the presenting manifestation. Histologically, BSLE lesions show a neutrophil-rich subepidermal split, and direct immunofluorescence demonstrates linear deposition of IgG and complement along the basement membrane zone (BMZ) (117). Immunologically, type I BSLE, the most common variant, is characterized by autoantibodies to type VII collagen, whereas type II BSLE is characterized by the absence of detectable anti-type VII collagen antibodies (117). Type VII collagen, encoded by *COL7A1*, is the major component of anchoring fibrils at the dermo-epidermal junction, and the corresponding antibodies recognize epitopes within the non-collagenous NC1 domain, including fibronectin-like regions (136).

A case report demonstrated that circulating anti-type VII collagen antibodies can precede the onset of clinical bullous lesions, with antibody levels increasing after lesion development and subsequently decreasing in parallel with cutaneous disease activity, supporting a potential pathogenic role in BSLE (137). Reactivity to other BMZ antigens, including BP230 and laminin-5/laminin-6, has also been described in BSLE, creating partial serological overlap with antigenic profiles observed in other autoimmune blistering diseases, including bullous pemphigoid and mucous membrane pemphigoid (138). Nonetheless, anti-type VII collagen remains the most characteristic and organ-relevant autoantibody in BSLE (117).

Overall, CLE illustrates how environmental stress and tissue-specific injury shape local autoantigenic landscapes in SLE. Anti-Ro/SSA and anti-La/SSB responses are closely linked to photosensitive SCLE, but their pathogenic relevance depends on ultraviolet-induced keratinocyte apoptosis, antigen redistribution, and local immune-complex deposition rather than strict skin specificity. RPLP0 and galectin-3 represent intracellular or injury-associated antigens that acquire cutaneous relevance in inflamed skin, whereas type VII collagen in BSLE provides a clearer example of a matrix-exposed structural target. Thus, cutaneous autoantigenicity spans apoptosis-driven exposure, inflammatory antigen redistribution, and direct targeting of dermo-epidermal junction components, supporting the organ-associated autoantigenic landscape model.

### 3.4 Vascular abnormalities in SLE

Vascular pathology in SLE is heterogeneous and includes immune-complex-mediated vasculitis, thrombotic vasculopathy, and accelerated atherosclerosis. These manifestations reflect the combined effects of chronic inflammation, dysregulated hemostasis, endothelial dysfunction, complement activation, aberrant neutrophil responses, and autoantibody-mediated endothelial or phospholipid-dependent perturbation (21, 139). In contrast to some renal or cutaneous manifestations, vascular disease in SLE is not defined by a single organ-restricted autoantigenic target. It reflects several overlapping humoral mechanisms, including loss or remodeling of protective natural antibody responses, endothelial-directed autoimmunity, NET-associated vascular injury, and thrombosis-promoting recognition of phospholipid-binding proteins.

Within this spectrum, accelerated atherosclerosis is shaped not only by pathogenic inflammation but also by altered humoral responses to oxidation-associated epitopes. Natural IgM produced by innate-like B-1 cells can bind oxidized lipid neoepitopes such as phosphorylcholine and may contribute to apoptotic-cell clearance and atheroprotection (140). In murine lupus, autoreactive B-1a cells expand but undergo class switching and redistribution during disease development, providing a plausible mechanism for loss of atheroprotective IgM and emergence of more pathogenic IgG responses (141). In humans with SLE, low levels of natural IgM against phosphorylcholine, a prototypic oxidation-associated epitope, are associated with vulnerable atherosclerotic plaques in SLE (142). Thus, vascular autoimmunity in SLE includes not only pathogenic autoantibody responses, but also the loss or remodeling of natural autoreactive IgM responses that may normally contribute to vascular protection.

Similarly, antibodies against apolipoprotein B-100 (apoB-100) peptides such as p45 and p210, which are linked to atheroprotection in non-lupus settings, are reduced in SLE and appear lowest in patients with established cardiovascular disease, supporting the possibility that reduced antibody responses to modified low-density lipoprotein (LDL)-related epitopes may contribute to vascular risk (143). By contrast, the clinical value of anti-oxidized LDL antibodies remains inconsistent: recent longitudinal data did not support a robust association with plaque occurrence, despite a correlation with femoral artery intima-media thickness (144). Additional lipid-related autoantigens relevant to SLE vasculopathy include lipoprotein lipase (LPL), a key enzyme in triglyceride metabolism. Anti-LPL IgG has been reported in patients with SLE and has been associated with dyslipidemia and inflammatory activity indices (145). These findings suggest that lipid-related autoantibody responses in SLE are heterogeneous: some may reflect loss of atheroprotective immunity, whereas others may mark dyslipidemia, inflammation, or vascular risk without necessarily acting as direct pathogenic mediators.

Several autoantibody systems may act more directly on the endothelium and are more plausibly linked to microvascular dysfunction and inflammatory vascular phenotypes. Anti-endothelial cell antibodies (AECA) in SLE represent a heterogeneous family of antibodies directed against multiple endothelial antigens rather than a single dominant target. Early characterization studies reported IgG AECA in 56 of 64 patients with SLE (88%) and showed broad reactivity across endothelial antigens (146). Their levels have been associated with higher disease activity and vascular manifestations such as digital vasculitis, and complement-fixing endothelial-binding IgG can induce C3 deposition and endothelial injury in experimental systems (147, 148). Because AECA comprise a heterogeneous antibody family, they are best interpreted as markers and potential mediators of endothelial activation rather than as a single antigen-defined biomarker (149).

Among better-defined endothelial targets, vascular endothelial cadherin (VE-cadherin/CDH5), a core adherens-junction protein required for endothelial barrier integrity, has emerged as a candidate autoantigen. SLE sera have been reported to recognize VE-cadherin, and patient IgG with high anti-VE-cadherin activity can disrupt endothelial junctional organization *in vitro* (150). This places VE-cadherin among plausible barrier-related endothelial targets, but its clinical significance in SLE vasculopathy requires further validation.

A more receptor-directed vascular mechanism is represented by anti-endothelin receptor type A (anti-ETAR) antibodies. These antibodies have been reported to be enriched in SLE-associated pulmonary arterial hypertension and have shown biologically relevant effects on endothelial barrier properties and smooth muscle proliferation in experimental systems (151). Within the broader vascular spectrum, anti-ETAR antibodies therefore illustrate receptor-directed vascular autoimmunity, although their strongest current relevance is to SLE-associated pulmonary arterial hypertension rather than to generalized SLE vasculopathy.

NETs provide a complementary mechanism linking autoimmunity to endothelial injury in SLE. In experimental studies, NETs from lupus low-density granulocytes contained increased externalized matrix metalloproteinase-9 (MMP-9). NET-associated MMP-9 promoted endothelial dysfunction and apoptosis by activating endothelial matrix metalloproteinase-2 (MMP-2). In addition, anti-MMP-9 IgG and MMP-9-containing immune complexes were identified in SLE sera, and these antibodies or immune complexes induced NETosis and enhanced NET-associated MMP-9 activity, supporting a feed-forward loop between NET formation and endothelial injury (152). This mechanism connects defective neutrophil regulation, autoantibody formation, and endothelial damage, but should be interpreted primarily as experimental evidence for vascular amplification rather than as an established clinical biomarker pathway.

Whereas AECA and related endothelial-directed antibodies are more closely linked to endothelial activation, barrier dysfunction, and vasculitic phenotypes, thrombotic vasculopathy in SLE is most robustly associated with aPL. These antibodies primarily recognize β2-glycoprotein I (β2GPI) and prothrombin when displayed on negatively charged phospholipid surfaces exposed during cell activation, apoptosis, or vascular injury. For β2GPI, antigen conformation and epitope specificity are critical, with antibodies directed against domain I, particularly around the G40–R43 region, showing the strongest association with thrombosis (153). Prothrombin becomes particularly antigenic when presented as a phosphatidylserine-prothrombin complex, and anti-phosphatidylserine/prothrombin (anti-PS/PT) antibodies are associated with lupus anticoagulant and with clinical APS manifestations (154–156). However, anti-PS/PT antibodies remain non-criteria biomarkers under the 2023 ACR/EULAR APS classification criteria, which include lupus anticoagulant, anticardiolipin, and anti-β2GPI antibodies and are intended for classification rather than diagnosis (19). Thus, aPL-mediated vascular disease represents one of the clearest examples in SLE in which antigen conformation, phospholipid exposure, and vascular injury converge to generate clinically relevant autoantibody effects.

Beyond canonical aPL, annexin-directed antibodies are plausible vascular modifiers because annexins sit at the interface of phospholipid exposure, coagulation, and fibrinolysis. Annexin A2 is an endothelial surface receptor that binds plasminogen, tissue plasminogen activator, and β2GPI, thereby supporting plasmin generation and fibrinolysis. Anti-annexin A2 antibodies are enriched in APS and in patients with SLE and thrombosis, and experimental studies have shown effects on tissue factor expression and annexin A2-supported plasmin generation, providing a plausible prothrombotic mechanism (157, 158). Annexin A5, by contrast, forms a two-dimensional anticoagulant shield over anionic phospholipids, and disruption of this layer has been proposed as a mechanism contributing to thrombosis and obstetric morbidity in aPL-positive disease. Hydroxychloroquine can experimentally protect this annexin A5 shield from aPL-mediated disruption (159, 160). In some cohorts, annexin A5 levels and anti-annexin A5 antibodies have been associated with coagulation-related parameters and disease severity in SLE (161). However, annexin-directed antibodies should be interpreted as plausible modifiers of phospholipid-dependent vascular pathology rather than as stand-alone diagnostic markers of SLE vasculopathy.

In summary, vascular abnormalities in SLE illustrate a complex autoantigenic landscape shaped by endothelial injury, lipid-related immune responses, NET-associated inflammation, and phospholipid-binding targets. Loss of natural IgM responses and reduced antibodies to apoB-100 peptides may contribute to accelerated atherosclerosis, whereas AECA, anti-VE-cadherin, and anti-ETAR antibodies reflect endothelial- or receptor-directed autoimmunity with variable evidence for direct pathogenicity. In parallel, NET-associated anti-MMP-9 responses may amplify endothelial injury, while aPL, anti-PS/PT, and annexin-directed antibodies link phospholipid exposure to thrombotic vasculopathy. Thus, vascular autoantigenicity in SLE is less defined by tissue-restricted antigens than by dynamic exposure of endothelial, lipid-related, phospholipid-binding, and coagulation-associated targets during inflammation, apoptosis, NETosis, and vascular injury, supporting the organ-associated autoantigenic landscape model.

### 3.5 Pulmonary involvement in SLE

Pulmonary involvement in SLE comprises a heterogeneous spectrum that includes pleuritis, acute lupus pneumonitis (ALP), diffuse alveolar hemorrhage (DAH), interstitial lung disease (ILD), and pulmonary arterial hypertension (PAH). It remains an important contributor to morbidity and mortality (162–164). Pleuropulmonary manifestations have been reported in approximately 50–70% of patients over the course of the disease, and up to 12% accumulate permanent lung damage within 10 years after diagnosis (162, 165). In contrast to some other organ manifestations of SLE, pulmonary disease is less readily attributable to a single dominant organ-associated autoantigen. Instead, lung injury usually reflects the interplay of systemic immune-complex-mediated inflammation, complement activation, endothelial injury, epithelial injury, barrier disruption, and, in selected phenotypes, vascular autoimmunity (162, 163). Thus, pulmonary involvement represents an important boundary case for the concept of organ-associated autoantigenic landscapes: some phenotypes appear primarily driven by systemic immune inflammation, whereas others show more defined links to vascular or epithelial autoimmunity. Shrinking lung syndrome and pulmonary embolism are also recognized pulmonary manifestations of SLE, but they are not discussed in detail here because reproducible antigen-defined pulmonary targets have not been clearly defined for these phenotypes.

Lupus pleuritis, the most common pulmonary manifestation of SLE, is generally regarded as an immune-complex- and complement-mediated serositis. Histopathologic descriptions include fibrinous pleuritis, mononuclear infiltration, and immunoglobulin and complement deposition within the pleura, while pleural fluid ANA positivity may support the diagnosis in the appropriate clinical context but is not specific (166, 167). Accordingly, pleuritis is best interpreted as a pulmonary manifestation of systemic immune-complex inflammation rather than as an antigen-defined lung-specific autoimmune process.

ALP, by contrast, is a rare but severe parenchymal complication that usually occurs in the setting of active systemic disease and may be associated with LN. Histologically, it is most often characterized by diffuse alveolar damage, with variable inflammatory infiltrates and edema (162, 164). Although anti-Ro/SSA antibodies have been reported in association with ALP in some studies, neither pleuritis nor ALP has been convincingly linked to a reproducible lung-specific autoantigen (168). Both phenotypes are therefore best framed as manifestations of systemic immune activation, complement-mediated inflammation, and parenchymal injury rather than as antigen-defined pulmonary autoimmunity.

DAH is a rare but life-threatening complication of SLE. Older series reported mortality rates as high as 60–75% (169), although outcomes appear to have improved in more recent cohorts (170). Clinically, DAH typically presents with acute dyspnea, hypoxemia, fever, cough, new diffuse alveolar infiltrates, and a rapid decline in hemoglobin (162, 171). Recent systematic review and meta-analytic data support an association between antiphospholipid antibody positivity and increased odds of DAH in SLE, particularly for lupus anticoagulant, anticardiolipin IgG, and concomitant APS, whereas anti-β2GPI IgG appears less consistently associated (172). These findings support the concept that DAH reflects injury at the alveolar-capillary interface, where capillaritis, immune-complex deposition, complement activation, endothelial injury, and the prothrombotic or proinflammatory effects of aPL may act synergistically to disrupt barrier integrity. In this context, aPL are not lung-specific autoantibodies, but they may contribute to pulmonary injury by targeting phospholipid-binding proteins exposed during endothelial activation, apoptosis, or microvascular damage.

Among pulmonary phenotypes, ILD shows the most plausible serological link to anti-Ro52 antibodies. A recent meta-analysis across autoimmune diseases supported an association between anti-Ro52 positivity and ILD, particularly with more severe phenotypes (173). A multicenter study of SLE-associated chronic ILD further characterized the SLE-ILD phenotype and reported frequent anti-SSA/Ro positivity, although it did not establish an anti-Ro52-specific association (174). Thus, anti-Ro52 should currently be viewed as a marker of autoimmune ILD susceptibility across connective tissue diseases rather than as a validated SLE-specific pulmonary autoantibody.

PAH represents the pulmonary phenotype with the strongest evidence for functionally relevant vascular autoantibodies in SLE. In SLE-associated PAH, anti-endothelin receptor type A (anti-ETAR) antibodies have been reported to be enriched and to exert biologically relevant effects in experimental systems (151). ETAR is a G-protein-coupled receptor expressed on vascular smooth muscle cells and endothelial cells, where it mediates endothelin-1-induced vasoconstriction, vascular remodeling, and proliferative signaling. Anti-ETAR antibodies may therefore act as stimulatory antibodies, enhancing smooth muscle proliferation, impairing endothelial barrier integrity, and upregulating PAH-related molecular programs. These effects can be attenuated by endothelin receptor antagonism. Anti-ETAR therefore represents one of the clearest antigen-associated examples in SLE of an autoantibody directed against a functionally relevant vascular target that may contribute directly to pulmonary vascular remodeling, although its clinical utility and disease specificity require further validation.

Overall, pulmonary involvement in SLE illustrates a spectrum from non-antigen-defined immune inflammation to more antigen-associated vascular pathology. Pleuritis and ALP are best understood as manifestations of systemic immune-complex-mediated inflammation, complement activation, and parenchymal injury, with limited evidence for reproducible lung-specific autoantigens. DAH reflects injury at the alveolar-capillary interface, where immune complexes, complement, capillaritis, endothelial injury, and aPL-associated microvascular injury may converge. ILD may be associated with anti-Ro52-positive autoimmune epithelial–immune dysregulation, but this relationship is not yet SLE-specific. By contrast, SLE-associated PAH provides a more defined example of receptor-directed vascular autoimmunity through anti-ETAR antibodies. Thus, pulmonary autoantigenicity in SLE is heterogeneous and often indirect, emerging primarily through vascular, epithelial, alveolar-capillary, and barrier injury rather than through a single lung-restricted autoantigen, supporting the organ-associated autoantigenic landscape model as a boundary case.

### 3.6 Hepatic involvement in SLE

Hepatic involvement in SLE is clinically heterogeneous and includes transient asymptomatic elevation of liver enzymes, lupus hepatitis, vascular liver injury, drug-induced hepatotoxicity, fatty liver disease, and true overlap with autoimmune liver diseases such as autoimmune hepatitis (AIH) or, more rarely, primary biliary cholangitis (PBC) (175–177). This heterogeneity makes the liver a distinct example of organ involvement in SLE, in which abnormal liver tests do not necessarily indicate primary lupus-driven hepatic autoimmunity. Lupus hepatitis is generally regarded as hepatic involvement attributable to SLE itself and should be distinguished from AIH, as the two conditions may share ANA positivity, hypergammaglobulinemia, and steroid responsiveness, yet differ in histology, serological associations, and long-term implications (176, 178). This distinction is clinically important because AIH usually implies a separate autoimmune liver disease requiring disease-specific evaluation and long-term management, whereas lupus hepatitis is often interpreted within the broader context of SLE activity.

The best-described serological association in lupus hepatitis is with anti-ribosomal P antibodies. In an early small case-control study, anti-ribosomal P antibodies were detected in all 6 patients with lupus hepatitis compared with 2 of 20 (10%) SLE controls without liver disease (reported OR 96; P = 0.0001) (179). Although these findings suggest a strong association, the small number of lupus-hepatitis cases limits the precision and generalizability of this estimate, and subsequent reviews have therefore regarded anti-ribosomal P antibodies as suggestive rather than diagnostically definitive markers of lupus hepatitis (129, 176, 179). Mechanistically, these antibodies can bind a membrane-associated P antigen-related epitope on hepatocytes and, *in vitro*, penetrate living hepatocytes and induce cellular dysfunction (180, 181). However, the *in vivo* pathogenicity of anti-ribosomal P antibodies remains incompletely established.

Older studies also described liver membrane autoantibodies (LMAs) in patients with SLE and abnormal liver function tests. LMAs were linked to disease activity and generally mild, transient hepatic abnormalities, supporting the concept that at least some liver test abnormalities in SLE may reflect immune-mediated but non-specific hepatic involvement (182). However, LMAs are not widely used as contemporary clinical biomarkers and should be discussed mainly as historical evidence for humoral recognition of hepatic membrane-associated targets in SLE.

A major diagnostic issue is that not all liver-related autoantibodies detected in patients with SLE indicate lupus hepatitis. Anti-smooth muscle antibodies, anti-liver–kidney microsomal antibodies, and anti-soluble liver antigen/liver–pancreas antibodies are more characteristic of AIH than of SLE-related hepatic inflammation. Conversely, antimitochondrial antibodies and PBC-associated nuclear antibodies such as anti-gp210 and anti-sp100 should raise suspicion for PBC overlap, particularly in the appropriate cholestatic biochemical context, rather than being interpreted as markers of primary lupus liver disease (178, 183). Likewise, atypical perinuclear antineutrophil cytoplasmic antibodies (p-ANCA) are better viewed as markers encountered in AIH- or primary sclerosing cholangitis (PSC)-spectrum disease than as indicators of hepatic SLE tropism (184).

Histology can be important when the diagnosis remains uncertain. AIH is typically characterized by interface hepatitis, whereas lupus hepatitis more often shows lobular hepatitis or non-specific lobular inflammation and generally lacks the classical AIH pattern (176, 178, 185). Accordingly, liver-associated autoantibodies in SLE should be interpreted in combination with liver biochemistry, medication exposure, viral testing when appropriate, metabolic risk factors, imaging, histology when available, and the broader autoimmune serological profile.

Vascular hepatic lesions should also be considered within the hepatic spectrum of SLE, particularly in patients with aPL, portal hypertensive features, or otherwise unexplained liver abnormalities. Nodular regenerative hyperplasia and other APS-related vascular complications are best interpreted as vascular hepatic injury rather than antigen-defined lupus hepatitis (176). This distinction is important because vascular injury, drug toxicity, fatty liver disease, and overlap with autoimmune liver disease may all coexist with SLE and can confound the interpretation of liver-associated autoantibodies.

In this context, hepatic involvement in SLE occupies an intermediate position between systemic immune injury, vascular injury, drug- or metabolism-related liver disease, and true organ-directed autoimmunity. Anti-ribosomal P antibodies provide the strongest serological link to lupus hepatitis, but their pathogenic role remains incompletely established. LMAs suggest that hepatic membrane-associated targets may become immunologically recognized in some patients, although their contemporary clinical utility is limited. Conversely, AIH- and PBC-associated autoantibodies should raise the possibility of overlap with autoimmune liver disease rather than be interpreted as markers of hepatic SLE tropism. Thus, hepatic autoantigenicity in SLE is best viewed as a diagnostic and mechanistic gray zone, requiring careful distinction between lupus-related hepatic inflammation, autoimmune liver disease overlap, vascular hepatic injury, medication toxicity, metabolic liver disease, and other non-immune causes of liver enzyme abnormalities.

## 4 Translating antigenic profiles into precision medicine: future therapeutic perspectives

The concept of organ-associated autoantigenic landscapes has therapeutic implications. If specific autoantibody–autoantigen axes prove pathogenic only within distinct tissue environments, therapeutic interventions should selectively target these pathways rather than relying on systemic immunosuppression. This provides a rationale for antigen-informed therapy in SLE, in which selected autoantibody systems may help guide approaches designed to block pathogenic antibody–antigen interactions, restore immune tolerance, or selectively eliminate autoreactive clones (186).

Currently, the most clinically advanced SLE immunotherapies lack antigen specificity. Notably, CD19-targeted CAR-T-cell therapy has demonstrated encouraging initial efficacy in severe, refractory SLE, achieving profound B-cell depletion, minimized serological activity, and clinical remission within small patient cohorts (187). B-cell maturation antigen (BCMA)-directed and combined CD19/BCMA-directed strategies extend this approach toward plasmablast and plasma-cell compartments, which may be particularly relevant when autoantibody production is sustained by differentiated antibody-secreting cells (188–190). These approaches, even when deploying next-generation CAR-T-based platforms such as switchable CARs (191) and *in vivo* CARs (192), demonstrate that a profound humoral immune reset can be therapeutically effective in SLE, but they remain broadly targeted interventions that deplete large B-cell populations rather than selectively eliminating B-cell clones with defined autoreactive specificities (193).

The antigen-specific approaches discussed herein potentially provide superior therapeutic precision by neutralizing defined pathogenic interactions. Specifically, peptide-based strategies illustrate two related but distinct antigen-informed approaches: blocking pathogenic antibody binding and restoring immune tolerance. Anti-dsDNA reactivity provides a clear example of antibody neutralization. The DWEYS peptide is recognized by a subset of anti-dsDNA antibodies that cross-react with NMDAR GluN2A/GluN2B subunits, linking this specificity to neuropsychiatric lupus mechanisms (69). DWEYS-derived decoys and the peptidomimetic FISLE-412 have been shown preclinically to interfere with anti-dsDNA/NMDAR antibody binding, while the D-form ALW peptide reduced renal immune deposition and injury in lupus-prone mice (194, 195). These studies support the principle that selected pathogenic antibody-antigen interactions may be neutralized without globally suppressing humoral immunity.

Another example of antigen-dependent pathway modulation in SLE involves tolerogenic peptides, which aim to regulate autoreactive immune responses rather than directly block antibodies. hCDR1/Edratide, a peptide derived from the complementarity-determining region of an anti-DNA antibody, was safe and well tolerated in a phase II SLE trial, although the primary endpoints were not met despite a secondary British Isles Lupus Assessment Group (BILAG)-based signal (196). P140/Lupuzor/IPP-201101, a phosphorylated peptide derived from U1 snRNP 70K, showed signals of clinical activity in phase IIb but has not become an established SLE therapy (197). Histone H4-derived peptides provide an additional preclinical example of nucleosomal tolerance induction in lupus-prone mice (198). While not yet applied to organ-specific SLE manifestations, this approach paves the way for utilizing distinct antigen-antibody pathways in future precision therapies.

This tolerance-oriented logic is also being extended to engineered regulatory T-cell approaches. A recent platform based on T-cell receptor-engineered regulatory T cells (TCR-Tregs) illustrates this direction. Ro60-specific TCR-Tregs recognized a Ro60-derived autoepitope presented by HLA-DRB1*03:01 and showed enhanced suppression of Ro60-reactive conventional CD4^+^ T-cell responses *in vitro* (199). Although developed in an anti-Ro/SSA-associated autoimmune context, this platform is conceptually relevant to Sjögren-overlap and anti-Ro-positive SLE phenotypes and should be viewed as an early antigen-specific tolerance strategy rather than an established therapy. It illustrates how identification of a disease-relevant autoepitope and its HLA restriction may be translated into an antigen-specific cellular tolerance strategy.

A more targeted strategy is selective elimination of autoreactive B cells using engineered receptors or bispecific molecules. Unlike conventional CD19- or BCMA-directed targeting, these platforms use an autoantigen, an autoantigen-derived epitope, or a shared autoreactive B-cell receptor feature as bait, with the goal of depleting disease-relevant autoreactive clones while sparing unrelated B cells.

Chimeric autoantibody receptor (CAAR) T cells display an autoantigen or autoantigen-derived domain on the effector-cell surface, enabling selective engagement of B cells whose B-cell receptors recognize that antigen. In LN, DNA-CAAR constructs incorporating antigens recognized by anti-dsDNA antibodies or other renal-relevant antigens selectively killed B cells bearing anti-dsDNA-reactive B-cell receptors *in vitro*, with DNA4-CAAR also reducing injury-associated features in a kidney organoid model (200). CAAR platforms based on natural killer (NK) cells, referred to as CAAR-NK platforms, apply a similar principle. For example, a La/SSB epitope-based CAAR-NK92MI platform selectively killed target cells bearing La/SSB-specific B-cell receptors *in vitro* (201). These approaches remain preclinical, but they directly translate autoantigen mapping into selective autoreactive B-cell targeting.

Antigen-targeted bispecific platforms follow the same conceptual direction. Bispecific autoantigen–T-cell engagers physically connect autoreactive B cells to cytotoxic T cells through an autoantigen domain and an anti-CD3 arm. A phospholipase A2 receptor (PLA2R)-based bispecific autoantigen–T-cell engagers in membranous nephropathy provide a platform example of autoantigen-specific B-cell depletion in autoimmune kidney disease, with conceptual relevance to SLE endotypes in which pathogenic B-cell receptor specificity can be validated (202). Similar principles are being explored for β2GPI/aPL-associated vascular autoimmunity through β2GPI-based bispecific autoantigen–T-cell engagers and β2GPI chimeric autoantigen receptor approaches designed to target anti-β2GPI B cells in antiphospholipid antibody-associated disease (203, 204).

Precision targeting need not rely exclusively on a single autoantigen. Idiotype-directed or repertoire-informed strategies instead exploit shared structural features of autoreactive B-cell receptors. The 9G4 idiotope, encoded by *IGHV4-34*, is enriched in active SLE and marks autoreactive B-cell compartments that can include anti-dsDNA- and anti-DNASE1L3-related responses. Anti-9G4 CAR T-cell and chimeric T-cell receptor approaches have been developed to selectively eliminate 9G4-positive autoreactive B cells in preclinical systems (205). In parallel, 9G4×CD3 T-cell-engaging bispecific antibodies have been reported as an off-the-shelf strategy to eliminate cells expressing 9G4-positive B-cell receptors while sparing non-9G4 B cells. In SLE peripheral blood mononuclear cell co-cultures, these molecules reduced the 9G4 B-cell compartment in a dose-dependent manner while largely preserving other antibody-secreting cells. This strategy may bridge broad B-cell depletion and single-antigen targeting by exploiting a shared autoreactive B-cell receptor feature, although its clinical efficacy and *in vivo* specificity remain to be established (206).

Antigen-informed therapy in SLE remains an emerging concept rather than an established clinical framework. Broad CD19-, BCMA-, and combined CD19/BCMA-directed cellular therapies currently have the strongest early clinical evidence but are not antigen-specific, whereas antibody decoys, tolerogenic peptides, antigen-specific regulatory cells, CAAR platforms, bispecific engagers, and idiotype-directed approaches remain largely preclinical or early translational. Their clinical development will require evidence that the targeted autoantibody or autoreactive B-cell population contributes directly to tissue injury, is accessible to therapeutic intervention, and defines a clinically identifiable patient endotype. Thus, autoantigenic profiling may ultimately support precision therapy and trial enrichment by linking clinical phenotype, autoantibody specificity, tissue context, pathogenic relevance, and treatment selection (207).

## 5 Discussion and conclusion

The broad autoantigen repertoire in SLE reflects the convergence of systemic mechanisms that generate and sustain autoreactivity, including defective clearance of apoptotic material, nucleic acid sensing, type I interferon signaling, B-cell tolerance failure, molecular mimicry, and epitope spreading (7, 8). However, systemic autoreactivity alone does not fully explain why individual patients develop distinct organ phenotypes. This clinical divergence suggests that local tissue vulnerability is shaped not only by the specificity of the autoreactive repertoire but also by antigen accessibility, tissue injury, immune-complex deposition, complement activation, vascular damage, barrier disruption, and microenvironment-specific inflammation (7, 20). Across major clinical manifestations, organ-relevant autoantigens can be organized into overlapping mechanistic classes that link systemic autoimmune processes to local tissue context. Rather than acting as passive bystanders of systemic inflammation, these antigens may serve as context-dependent points of convergence through which systemic autoreactivity is translated into local inflammatory and tissue-injury pathways (**Figure 3**).

**Figure 3:**
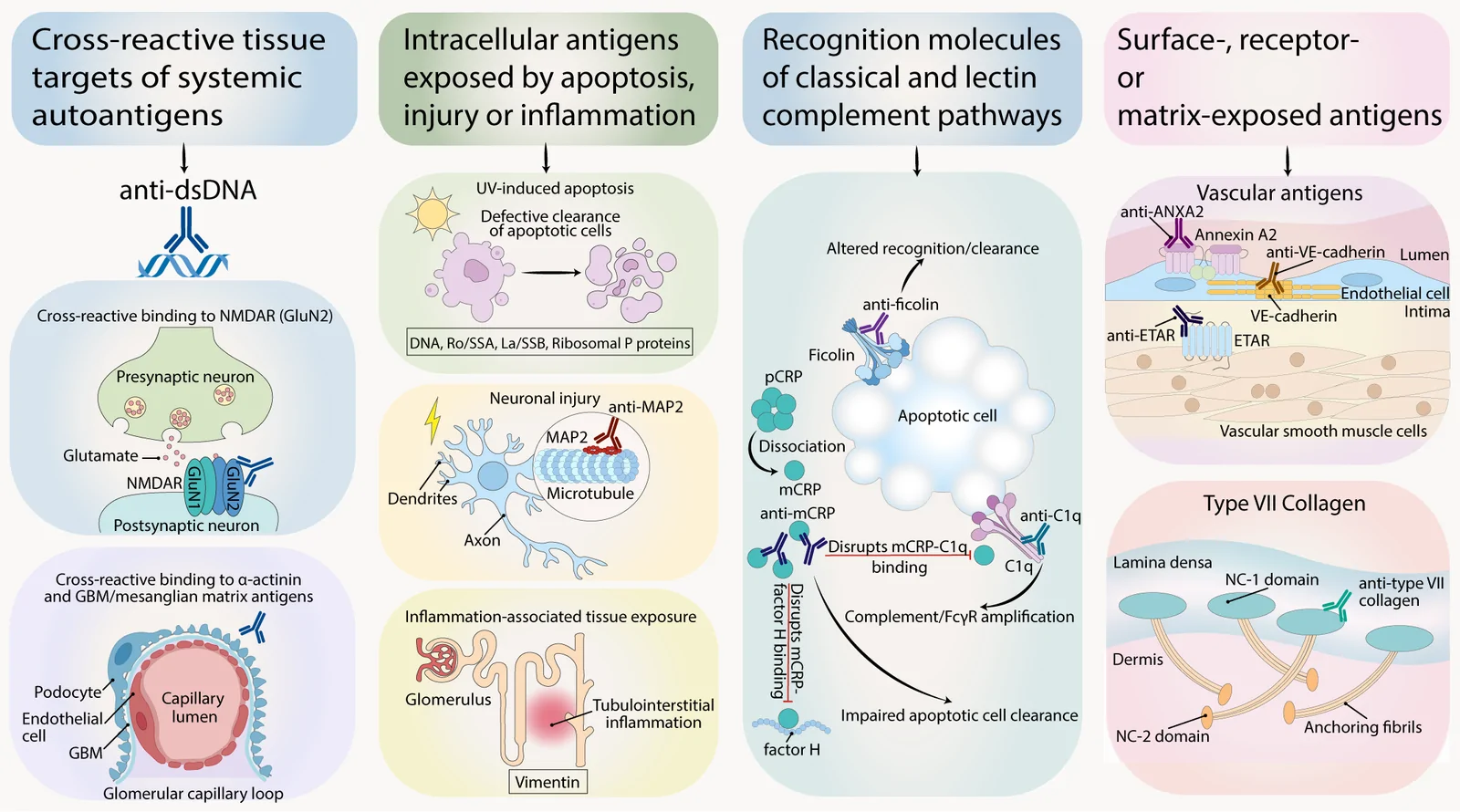
Mechanistic classes of organ-relevant autoantigens in systemic lupus erythematosus. Organ-relevant autoantigens in systemic lupus erythematosus can be conceptualized as overlapping mechanistic classes rather than strictly organ-specific targets. Cross-reactive tissue targets of systemic autoantibodies are exemplified by anti-dsDNA cross-reactivity with NMDAR/GluN2 in the central nervous system and with α-actinin or GBM/mesangial matrix components in LN. A second class comprises intracellular antigens that become immunologically accessible after apoptosis, tissue injury, or inflammation, including Ro/SSA, La/SSB, ribosomal P proteins, MAP2, and vimentin. A third class includes recognition molecules of the classical and lectin complement pathways, such as C1q, modified or monomeric CRP, ficolin-2, and ficolin-3; autoimmunity against these molecules may impair clearance and amplify immune-complex-driven complement activation. Surface-, receptor-, or matrix-exposed functional targets include ETAR, VE-cadherin, annexin A2, and type VII collagen, which may affect vascular tone, barrier integrity, fibrinolysis, or tissue architecture. These classes are not mutually exclusive and may overlap depending on tissue context, inflammation, antigen accessibility, and disease stage. ANXA2, annexin A2; C1q, complement component C1q; CRP, C-reactive protein; ETAR, endothelin receptor type A; FcγR, Fcγ receptor; GBM, glomerular basement membrane; mCRP, monomeric or modified C-reactive protein; MAP2, microtubule-associated protein 2; NMDAR, N-methyl-D-aspartate receptor; GluN2, glutamate ionotropic receptor NMDA-type subunit 2; VE-cadherin, vascular endothelial cadherin.

First, cross-reactive tissue targets of systemic autoantibodies are exemplified by anti-dsDNA-related reactivity with NMDAR/GluN2 subunits in the CNS and with α-actinin, heparan sulfate, or matrix-associated structures in the kidney (69, 102, 106). Second, intracellular antigens exposed during apoptosis, NET formation, inflammation, or tissue injury, including Ro/SSA, La/SSB, ribosomal P proteins, MAP2, α-enolase, annexin A1, and vimentin, may become immunologically accessible in inflamed tissues (20, 108, 111). Third, recognition molecules of the classical and lectin complement pathways, including C1q, modified or monomeric CRP, ficolin-2, and ficolin-3, may become autoantigenic targets and potentially amplify immune-complex handling defects, particularly in LN (16, 94, 98–100). Fourth, surface, receptor, matrix, phospholipid-binding, and extracellular targets, including ETAR, VE-cadherin, type VII collagen, annexin A2, annexin A5, β2GPI, and prothrombin, may acquire relevance because they are accessible at sites of vascular activation, coagulation, barrier injury, or tissue remodeling (19, 117, 151).

Importantly, this framework does not imply that autoantibodies alone determine organ damage. Autoantibody-mediated effects are embedded in broader pathogenic networks that include cellular immunity, dysregulated cytokine signaling, complement activation, vascular injury, and tissue-resident inflammatory pathways (7, 8). The interaction between a specific autoantigen and its corresponding autoantibody should therefore not be interpreted as a stand-alone cause of tissue injury, but as one component of a local inflammatory and effector network. These mechanistic classes are not mutually exclusive: individual autoantigens may shift between categories depending on tissue context, disease phase, cellular localization, post-translational modification, antigen conformation, and the surrounding inflammatory microenvironment.

Autoantibodies associated with several organ systems provide an important test of this framework. Organ association should not be equated with strict organ specificity: the same circulating autoantibody or autoantibody system may participate in different organ-associated landscapes when its cognate or cross-reactive targets become accessible under distinct tissue conditions. Anti-dsDNA-related reactivity illustrates this principle, because selected antibodies may acquire renal relevance through recognition of glomerular or matrix-associated targets and CNS relevance through cross-reactivity with GluN2-containing NMDARs when the blood–brain barrier permits antibody access (69, 71, 102, 106). The anti-Ro/SSA system provides a second informative example. Its relevance in photosensitive cutaneous lupus is associated with ultraviolet-induced keratinocyte apoptosis and antigen redistribution, whereas fetal cardiac injury appears to depend additionally on transplacental antibody access and a developmentally susceptible tissue context (125–128). These examples suggest that organ association emerges from the combination of antibody specificity, antigen accessibility or conformation, tissue access, and local effector conditions rather than from antibody specificity alone.

Accordingly, organ-associated autoantigenicity in SLE should be understood as dynamic and context-dependent rather than as a fixed property of individual antigens. Organ-associated autoantibodies do not represent a single biological class. Some may contribute directly to tissue injury, some function mainly as diagnostic or prognostic biomarkers, others may reflect secondary tissue damage or local immune activation, and many remain unresolved associations requiring further validation.

Autoimmune cytopenias represent a related boundary case for this framework because the relevant targets are located on circulating blood cells rather than within a fixed organ. In SLE-associated autoimmune hemolytic anemia, erythrocyte-bound immunoglobulins may promote Fc receptor- and complement-dependent clearance. Lupus thrombocytopenia is more heterogeneous and may involve antiplatelet antibodies, antiphospholipid antibody-associated mechanisms, and peripheral platelet destruction; normal or increased megakaryocyte numbers in many bone-marrow samples support the latter mechanism in a substantial subset of patients. In a multicenter prospective CSTAR cohort of 350 patients with SLE-associated thrombocytopenia, 194 (55.4%) were aPL-positive. These patients had lower baseline platelet counts and a higher relapse rate than aPL-negative patients (58.2% vs 44.2%), while age was similar between the groups, supporting aPL status as a clinically relevant modifier of the hematological phenotype rather than a simple demographic association. However, not all cytopenias in SLE are autoantibody-mediated, and inflammation, infection, treatment effects, or impaired hematopoiesis may also contribute. Hematological relevance should therefore be inferred only when clinical and laboratory evidence supports immune-mediated destruction, rather than from reduced blood-cell counts or autoantibody detection alone (208–211).

Host-level determinants provide an additional layer of heterogeneity within this framework. Factors such as age, sex, genetic background, comorbidities, and treatment exposure may modify tissue susceptibility and the clinical expression of systemic autoreactivity. Longitudinal studies support this concept: predictors of damage differ across organ domains in SLE (212), while younger age at diagnosis and male sex, together with serological activity, have been associated with subsequent kidney involvement (213). These host-level characteristics should therefore be viewed as modifiers of the interaction between systemic autoreactivity and local tissue conditions rather than as independent determinants of organ tropism.

The framework also generates several testable expectations. First, among patients with the same circulating autoantibody specificity, differences in organ involvement would be expected to be associated with differences in tissue access, local antigen availability, or effector activation. Second, markers of these local conditions would be expected to add predictive information beyond serum autoantibody status or titer alone. Third, organ-specific activity may emerge or resolve without a corresponding change in autoantibody specificity when the relevant tissue context changes. These expectations could be evaluated by comparing serologically similar patients with discordant organ phenotypes and through longitudinal paired blood–CSF or blood–tissue studies performed before, during, and after organ-specific disease activity. They are proposed as testable hypotheses rather than established conclusions.

The strength of evidence differs substantially across organ systems. LN provides one of the clearest examples of organ-focused autoantigenicity because circulating autoantibody profiles, renal histology, immune deposits, complement activation, and local humoral responses can be integrated (14, 108). By contrast, NPSLE and pulmonary involvement illustrate more heterogeneous and attribution-sensitive settings, in which autoantibodies, vascular injury, barrier disruption, infection, treatment effects, and non-lupus processes may overlap (62, 164). Hepatic involvement is similarly complex because lupus hepatitis, overlap with autoimmune liver disease, antiphospholipid-related vascular injury, medication toxicity, and metabolic liver disease must be distinguished before liver-associated autoantibodies can be interpreted as evidence of hepatic SLE tropism (176, 178).

Several limitations should be acknowledged. Much of the evidence linking autoantibodies to organ manifestations in SLE is associative and derives from cross-sectional studies, small cohorts, selected patient populations, or experimental models that may not fully reproduce human disease heterogeneity. Differences in assay platforms, antigen preparation, epitope specificity, antibody isotype, clinical phenotype definitions, treatment exposure, and disease duration further complicate comparisons across studies. Also, standardized validation of candidate biomarkers across independent cohorts remains limited, restricting comparison of their diagnostic and prognostic performance and their translation into clinical practice. In addition, few studies integrate circulating autoantibody profiles with longitudinal tissue-based validation, local immune-cell repertoires, local antigen accessibility, local effector activation, complement activation, transcriptomic signatures, imaging, and clinical outcomes over time. This limits direct testing of whether tissue-specific contextual markers improve organ attribution beyond circulating autoantibody profiles alone. Future studies should therefore distinguish more explicitly between pathogenic autoantibodies, prognostic biomarkers, secondary injury-associated responses, and unresolved associations. Integration of paired blood and tissue BCR and TCR repertoire analyses with spatial profiling and immunopeptidomics may help identify self-antigens that are locally presented and recognized within affected organs. However, reproducible shared pathogenic lymphocyte clonotypes have not yet been consistently identified in SLE (214, 215). The acceleration of the development of molecular diagnostic tools for a subtype of SLE with specific organ involvement will likely depend on the integration of new high-throughput technologies into a unified multi-level approach. Single-cell RNA sequencing paired with B-cell receptor sequencing could offer an unprecedented capability to define the repertoires of autoreactive B cells within compromised microenvironments. This technology may reveal how pathogenic, tissue-infiltrating clones migrate into specific target organs, such as the kidneys in LN or skin lesions in CLE (216–218). Systematically clustering these autoreactive BCR repertoires across diverse SLE subtypes might help address current diagnostic challenges. Furthermore, comparing tissue-resident B cells with peripheral blood signatures could be helpful for immunometric databases, thereby assisting in the prediction of specific organ injury and the optimization of targeted therapies. Nowadays, as whole-genome sequencing (WGS) becomes more accessible for clinical translation, it might provide a scalable method to screen individual patients for genetic variants that could predispose them to certain organ vulnerabilities. For instance, WGS could potentially identify genetic variants responsible for blood-brain barrier permeability in NPSLE, or those predisposing to complement system deficiencies in severe LN. This genomic risk profile likely intersects with cellular pathology. Age-associated B cells, which expand in SLE patients and persist within inflamed end-organs, express high levels of GWAS-identified SLE risk genes, potentially supporting the view that these cells actively contribute to localized tissue destruction (219). The “autoantibody reactome”, the multidimensional repertoire of antibody reactivities against self-antigens, offers a scalable systemic layer that can connect systemic autoreactivity to tissue pathology across autoimmune disorders (220). Combining genetic backgrounds with immunological assessments as a multi-omics approach should aid better diagnosis and prediction of organ targeting in SLE, supporting the development of clinically validated biomarker panels for risk prediction and precision therapeutic selection.

Overall, the concept of organ-associated autoantigenic landscapes helps explain how systemic autoimmunity can be translated into clinically heterogeneous organ phenotypes without requiring strictly organ-specific or organ-restricted autoantigens. Rather than treating organ-associated autoantibodies as isolated biomarkers, this framework places them within tissue-specific contexts of antigen exposure, immune-complex handling, complement activation, vascular injury, barrier dysfunction, and local inflammation. Recognizing these context-dependent autoantigen classes may refine biomarker interpretation, improve patient stratification, and support future antigen-informed precision approaches in SLE.

## References

[1] Tsokos GC . Systemic lupus erythematosus. N Engl J Med. (2011) 365:2110–21. doi: 10.1056/NEJMra1100359 22129255

[2] Lisnevskaia L Murphy G Isenberg D . Systemic lupus erythematosus. Lancet. (2014) 384:1878–88. doi: 10.1016/S0140-6736(14)60128-8 24881804

[3] Kaul A Gordon C Crow MK Touma Z Urowitz MB Van Vollenhoven R . Systemic lupus erythematosus. Nat Rev Dis Primers. (2016) 2:16039. doi: 10.1038/nrdp.2016.39 27306639

[4] Matusiewicz A Paś A Wiktorzak S Olesińska M . Redefining difficult-to-treat systemic lupus erythematosus: Biomarkers of molecular refractoriness beyond clinical failure. Int J Mol Sci. (2026) 27:4026. doi: 10.3390/ijms27094026 42123602 PMC13163840

[5] Nasonov EL Soloviev SK Arshinov AV . Systemic lupus erythematosus: history and modernity. Nauchno-Prakt Revmatol. (2022) 60:397–412. doi: 10.47360/1995-4484-2022-397-412

[6] Mahajan A Herrmann M Muñoz LE . Clearance deficiency and cell death pathways: A model for the pathogenesis of SLE. Front Immunol. (2016) 7:35. doi: 10.3389/fimmu.2016.00035 26904025 PMC4745266

[7] Tsokos GC . Autoimmunity and organ damage in systemic lupus erythematosus. Nat Immunol. (2020) 21:605–14. doi: 10.1038/s41590-020-0677-6 32367037 PMC8135909

[8] Gómez-Bañuelos E Fava A Andrade F . An update on autoantibodies in systemic lupus erythematosus. Curr Opin Rheumatol. (2023) 35:61–7. doi: 10.1097/BOR.0000000000000922 36695053 PMC9881844

[9] Pisetsky DS . Anti-DNA antibodies — quintessential biomarkers of SLE. Nat Rev Rheumatol. (2016) 12:102–10. doi: 10.1038/nrrheum.2015.151 26581343

[10] Shuster AM Gololobov GV Kvashuk OA Bogomolova AE Smirnov IV Gabibov AG . DNA hydrolyzing autoantibodies. Science. (1992) 256:665–7. doi: 10.1126/science.1585181 1585181

[11] Gololobov GV Chernova EA Schourov DV Smirnov IV Kudelina IA Gabibov AG . Cleavage of supercoiled plasmid DNA by autoantibody Fab fragment: Application of the flow linear dichroism technique. Proc Natl Acad Sci USA. (1995) 92:254–7. doi: 10.1073/pnas.92.1.254 7816827 PMC42856

[12] Lomakin Y Kudriaeva A Kostin N Terekhov S Kaminskaya A Chernov A . Diagnostics of autoimmune neurodegeneration using fluorescent probing. Sci Rep. (2018) 8:12679. doi: 10.1038/s41598-018-30938-0 30139963 PMC6107501

[13] Nasonov EL Reshetnyak TM Solovyev SK Popkova TV . Systemic lupus erythematosus and antiphospholipid syndrome: Past, present, future. Ter Arkh. (2023) 95:365–74. doi: 10.26442/00403660.2023.05.202246 38158987

[14] Bruschi M Angeletti A Prunotto M Meroni PL Ghiggeri GM Moroni G . A critical view on autoantibodies in lupus nephritis: Concrete knowledge based on evidence. Autoimmun Rev. (2024) 23:103535. doi: 10.1016/j.autrev.2024.103535 38552995

[15] Parodis I Nikolopoulos D Brylid A Hultgren O Alarcón-Riquelme ME Sjöwall C . Old and new autoantibodies in systemic lupus erythematosus. Expert Rev Clin Immunol. (2026) 22:19–29. doi: 10.1080/1744666X.2026.2625964 41636702

[16] Calatroni M Moroni G Conte E Stella M Reggiani F Ponticelli C . Anti-C1q antibodies: A biomarker for diagnosis and management of lupus nephritis. A narrative review. Front Immunol. (2024) 15:1410032. doi: 10.3389/fimmu.2024.1410032 38938561 PMC11208682

[17] Lee LA Roberts CM Frank MB McCubbin VR Reichlin M . The autoantibody response to Ro/SSA in cutaneous lupus erythematosus. Arch Dermatol. (1994) 130:1262–8. doi: 10.1001/archderm.1994.01690100046006 7944507

[18] Stull C Sprow G Werth VP . Cutaneous involvement in systemic lupus erythematosus: A review for the rheumatologist. J Rheumatol. (2023) 50:27–35. doi: 10.3899/jrheum.220089 36109075 PMC10152495

[19] Barbhaiya M Zuily S Naden R Hendry A Manneville F Amigo M-C . The 2023 ACR/EULAR antiphospholipid syndrome classification criteria. Arthritis Rheumatol. (2023) 75:1687–702. doi: 10.1002/art.42624 37635643

[20] Casciola-Rosen LA Anhalt G Rosen A . Autoantigens targeted in systemic lupus erythematosus are clustered in two populations of surface structures on apoptotic keratinocytes. J Exp Med. (1994) 179:1317–30. doi: 10.1084/jem.179.4.1317 7511686 PMC2191465

[21] Moschetti L Piantoni S Vizzardi E Sciatti E Riccardi M Franceschini F . Endothelial dysfunction in systemic lupus erythematosus and systemic sclerosis: A common trigger for different microvascular diseases. Front Med (Lausanne). (2022) 9:849086. doi: 10.3389/fmed.2022.849086 35462989 PMC9023861

[22] Moser KL Kelly JA Lessard CJ Harley JB . Recent insights into the genetic basis of systemic lupus erythematosus. Genes Immun. (2009) 10:373–9. doi: 10.1038/gene.2009.39 19440199 PMC3144759

[23] Graham RR Kozyrev SV Baechler EC Reddy MVPL Plenge RM Bauer JW . A common haplotype of interferon regulatory factor 5 (IRF5) regulates splicing and expression and is associated with increased risk of systemic lupus erythematosus. Nat Genet. (2006) 38:550–5. doi: 10.1038/ng1782 16642019

[24] James JA Gross T Scofield RH Harley JB . Immunoglobulin epitope spreading and autoimmune disease after peptide immunization: Sm B/B’-derived PPPGMRPP and PPPGIRGP induce spliceosome autoimmunity. J Exp Med. (1995) 181:453–61. doi: 10.1084/jem.181.2.453 7530756 PMC2191871

[25] McClain MT Heinlen LD Dennis GJ Roebuck J Harley JB James JA . Early events in lupus humoral autoimmunity suggest initiation through molecular mimicry. Nat Med. (2005) 11:85–9. doi: 10.1038/nm1167 15619631

[26] Yurasov S Wardemann H Hammersen J Tsuiji M Meffre E Pascual V . Defective B cell tolerance checkpoints in systemic lupus erythematosus. J Exp Med. (2005) 201:703–11. doi: 10.1084/jem.20042251 15738055 PMC2212839

[27] Crow MK . Type I interferon in the pathogenesis of lupus. J Immunol. (2014) 192:5459–68. doi: 10.4049/jimmunol.1002795 24907379 PMC4083591

[28] Fillatreau S Manfroi B Dörner T . Toll-like receptor signalling in B cells during systemic lupus erythematosus. Nat Rev Rheumatol. (2021) 17:98–108. doi: 10.1038/s41584-020-00544-4 33339987 PMC7747191

[29] Tipton CM Hom JR Fucile CF Rosenberg AF Sanz I . Understanding B‐cell activation and autoantibody repertoire selection in systemic lupus erythematosus: A B‐cell immunomics approach. Immunol Rev. (2018) 284:120–31. doi: 10.1111/imr.12660 29944759 PMC6022284

[30] Muñoz LE Lauber K Schiller M Manfredi AA Herrmann M . The role of defective clearance of apoptotic cells in systemic autoimmunity. Nat Rev Rheumatol. (2010) 6:280–9. doi: 10.1038/nrrheum.2010.46 20431553

[31] Al-Azab M Idiiatullina E Liu Z Lin M Hrovat-Schaale K Xian H . Genetic variants in UNC93B1 predispose to childhood-onset systemic lupus erythematosus. Nat Immunol. (2024) 25:969–80. doi: 10.1038/s41590-024-01846-5 38831104 PMC11147776

[32] Vinuesa CG Shrotri M Rahman A . TLR7 in systemic lupus erythematosus: Genetics and emerging therapies. Nat Rev Rheumatol. (2026) 22:479–95. doi: 10.1038/s41584-026-01388-0 42321474

[33] Hakkim A Fürnrohr BG Amann K Laube B Abed UA Brinkmann V . Impairment of neutrophil extracellular trap degradation is associated with lupus nephritis. Proc Natl Acad Sci USA. (2010) 107:9813–8. doi: 10.1073/pnas.0909927107 20439745 PMC2906830

[34] Reshetnyak T Nurbaeva K Ptashnik I Kudriaeva A Belogurov A Lila A . Markers of NETosis in patients with systemic lupus erythematosus and antiphospholipid syndrome. Int J Mol Sci. (2023) 24:9210. doi: 10.3390/ijms24119210 37298160 PMC10252548

[35] Kaplan MJ . Exploring the role of neutrophil extracellular traps in systemic lupus erythematosus: A clinical case study and comprehensive review. Arthritis Rheumatol. (2025) 77:247–52. doi: 10.1002/art.43019 39402725 PMC11865695

[36] Sisirak V Sally B D’Agati V Martinez-Ortiz W Özçakar ZB David J . Digestion of chromatin in apoptotic cell microparticles prevents autoimmunity. Cell. (2016) 166:88–101. doi: 10.1016/j.cell.2016.05.034 27293190 PMC5030815

[37] Al-Mayouf SM Sunker A Abdwani R Abrawi SA Almurshedi F Alhashmi N . Loss-of-function variant in DNASE1L3 causes a familial form of systemic lupus erythematosus. Nat Genet. (2011) 43:1186–8. doi: 10.1038/ng.975 22019780

[38] Kisla Ekinci RM Balci S Ozcan D Atmis B Bisgin A . Monogenic lupus due to DNASE1L3 deficiency in a pediatric patient with urticarial rash, hypocomplementemia, pulmonary hemorrhage, and immune-complex glomerulonephritis. Eur J Med Genet. (2021) 64:104262. doi: 10.1016/j.ejmg.2021.104262 34161863

[39] Ueki M Takeshita H Fujihara J Iida R Yuasa I Kato H . Caucasian-specific allele in non-synonymous single nucleotide polymorphisms of the gene encoding deoxyribonuclease I-like 3, potentially relevant to autoimmunity, produces an inactive enzyme. Clin Chim Acta. (2009) 407:20–4. doi: 10.1016/j.cca.2009.06.022 19559017

[40] Coke LN Wen H Comeau M Ghanem MH Shih A Metz CN . Arg206Cys substitution in DNASE1L3 causes a defect in DNASE1L3 protein secretion that confers risk of systemic lupus erythematosus. Ann Rheum Dis. (2021) 80:782–7. doi: 10.1136/annrheumdis-2020-218810 33455918 PMC8142439

[41] Hartl J Serpas L Wang Y Rashidfarrokhi A Perez OA Sally B . Autoantibody-mediated impairment of DNASE1L3 activity in sporadic systemic lupus erythematosus. J Exp Med. (2021) 218:e20201138. doi: 10.1084/jem.20201138 33783474 PMC8020718

[42] Rönnblom L Leonard D . Interferon pathway in SLE: One key to unlocking the mystery of the disease. Lupus Sci Med. (2019) 6:e000270. doi: 10.1136/lupus-2018-000270 31497305 PMC6703304

[43] Oke V Gunnarsson I Dorschner J Eketjäll S Zickert A Niewold TB . High levels of circulating interferons type I, type II and type III associate with distinct clinical features of active systemic lupus erythematosus. Arthritis Res Ther. (2019) 21:107. doi: 10.1186/s13075-019-1878-y 31036046 PMC6489203

[44] Sigurdsson S Göring HHH Kristjansdottir G Milani L Nordmark G Sandling JK . Comprehensive evaluation of the genetic variants of interferon regulatory factor 5 (IRF5) reveals a novel 5 bp length polymorphism as strong risk factor for systemic lupus erythematosus. Hum Mol Genet. (2008) 17:872–81. doi: 10.1093/hmg/ddm359 18063667

[45] Fu Q Zhao J Qian X Wong JLH Kaufman KM Yu CY . Association of a functional IRF7 variant with systemic lupus erythematosus. Arthritis Rheum. (2011) 63:749–54. doi: 10.1002/art.30193 21360504 PMC3063317

[46] Lessard CJ Adrianto I Ice JA Wiley GB Kelly JA Glenn SB . Identification of IRF8, TMEM39A, and IKZF3-ZPBP2 as susceptibility loci for systemic lupus erythematosus in a large-scale multiracial replication study. Am J Hum Genet. (2012) 90:648–60. doi: 10.1016/j.ajhg.2012.02.023 22464253 PMC3322228

[47] Zhou T Zhu X Ye Z Wang Y-F Yao C Xu N . Lupus enhancer risk variant causes dysregulation of IRF8 through cooperative lncRNA and DNA methylation machinery. Nat Commun. (2022) 13:1855. doi: 10.1038/s41467-022-29514-y 35388006 PMC8987079

[48] Remmers EF Plenge RM Lee AT Graham RR Hom G Behrens TW . STAT4 and the risk of rheumatoid arthritis and systemic lupus erythematosus. N Engl J Med. (2007) 357:977–86. doi: 10.1056/NEJMoa073003 17804842 PMC2630215

[49] Sigurdsson S Nordmark G Göring HHH Lindroos K Wiman A-C Sturfelt G . Polymorphisms in the tyrosine kinase 2 and interferon regulatory factor 5 genes are associated with systemic lupus erythematosus. Am J Hum Genet. (2005) 76:528–37. doi: 10.1086/428480 15657875 PMC1196404

[50] Kozyrev SV Abelson A-K Wojcik J Zaghlool A Linga Reddy MVP Sanchez E . Functional variants in the B-cell gene BANK1 are associated with systemic lupus erythematosus. Nat Genet. (2008) 40:211–6. doi: 10.1038/ng.79 18204447

[51] Cancro MP D’Cruz DP Khamashta MA . The role of B lymphocyte stimulator (BLyS) in systemic lupus erythematosus. J Clin Invest. (2009) 119:1066–73. doi: 10.1172/JCI38010 19411764 PMC2673851

[52] Zhu D-D Castrillon C Akama-Garren E Carroll MC . Germinal-centre and extrafollicular B cell pathways in systemic lupus erythematosus. Nat Rev Rheumatol. (2026) 22:285–301. doi: 10.1038/s41584-026-01365-7 41946971 PMC13217407

[53] Tariq F Charavanmuttu Y Labiba K Wincup C . Aberrant B cell responses as drivers of autoantibody generation and epitope diversification in SLE pathogenesis. Front Immunol. (2026) 16:1731285. doi: 10.3389/fimmu.2025.1731285 41583477 PMC12827690

[54] Poole BD Scofield RH Harley JB James JA . Epstein-Barr virus and molecular mimicry in systemic lupus erythematosus. Autoimmunity. (2006) 39:63–70. doi: 10.1080/08916930500484849 16455583

[55] Ovchinnikova LA Eliseev IE Dzhelad SS Simaniv TO Klimina KM Ivanova M . High heterogeneity of cross-reactive immunoglobulins in multiple sclerosis presumes combining of B-cell epitopes for diagnostics: a case-control study. Front Immunol. (2024) 15:1401156. doi: 10.3389/fimmu.2024.1401156 39669579 PMC11634884

[56] Zhao Z Ren J Dai C Kannapell CC Wang H Gaskin F . Nature of T cell epitopes in lupus antigens and HLA-DR determines autoantibody initiation and diversification. Ann Rheum Dis. (2019) 78:380–90. doi: 10.1136/annrheumdis-2018-214125 30254034 PMC6377330

[57] Lum FM Sattarnezhad N Ho PP Orr N Robinson WH Lanz TV . Multiple molecular mimics in Epstein Barr Nuclear Antigen-1, and the pathogenesis of multiple sclerosis. Proc Natl Acad Sci USA. (2026) 123:e2519445123. doi: 10.1073/pnas.2519445123 41790925 PMC12994170

[58] Hsieh S-J Tsai T-H Lin J-H Lin T-Y Chang F-L Chiang C-W . Characterization of anti-EBNA-1 antibodies and exploration of their molecular mimicry potential in an EBV-infected Sjögren’s syndrome patient. Biochem Biophys Res Commun. (2024) 735:150839. doi: 10.1016/j.bbrc.2024.150839 39427375

[59] Bruschi M Angeletti A Kajana X Moroni G Sinico RA Fredi M . Evidence for charge-based mimicry in anti dsDNA antibody generation. J Autoimmun. (2022) 132:102900. doi: 10.1016/j.jaut.2022.102900 36087539

[60] Monneaux F Muller S . Epitope spreading in systemic lupus erythematosus: Identification of triggering peptide sequences. Arthritis Rheum. (2002) 46:1430–8. doi: 10.1002/art.10263 12115171

[61] Arbuckle MR McClain MT Rubertone MV Scofield RH Dennis GJ James JA . Development of autoantibodies before the clinical onset of systemic lupus erythematosus. N Engl J Med. (2003) 349:1526–33. doi: 10.1056/NEJMoa021933 14561795

[62] Legge AC Hanly JG . Recent advances in the diagnosis and management of neuropsychiatric lupus. Nat Rev Rheumatol. (2024) 20:712–28. doi: 10.1038/s41584-024-01163-z 39358609

[63] Li LY Höltje M Rasmussen HF Halle L Mayrhofer M Blüthner M . Binding of established antinuclear antibodies to neurons depends on tissue fixation and underlying autoantigens. Front Immunol. (2025) 16:1674907. doi: 10.3389/fimmu.2025.1674907 41142797 PMC12549672

[64] Sato T Fujii T Yokoyama T Fujita Y Imura Y Yukawa N . Anti-U1 RNP antibodies in cerebrospinal fluid are associated with central neuropsychiatric manifestations in systemic lupus erythematosus and mixed connective tissue disease. Arthritis Rheum. (2010) 62:3730–40. doi: 10.1002/art.27700 20722023

[65] Abdel-Nasser AM Ghaleb RM Mahmoud JA Khairy W Mahmoud RM . Association of anti-ribosomal P protein antibodies with neuropsychiatric and other manifestations of systemic lupus erythematosus. Clin Rheumatol. (2008) 27:1377–85. doi: 10.1007/s10067-008-0921-1 18481154

[66] Lisitsyna TA Reshetnyak TM Diatroptov ME Avdeeva AS Nurbaeva KS Abramkin AA . The clinical significance of antibodies to ribosomal protein P (a-Rib-P) in patients with systemic lupus erythematosus and antiphospholipid syndrome. Nauchno-Prakt Revmatol. (2026) 64:279–87. doi: 10.47360/1995-4484-2026-279-287

[67] Manikuppam P Prakash JAJ Yadav B Mathew J . Beyond paraneoplastic neurological syndromes: Anti-neuronal antibodies in neuropsychiatric systemic lupus erythematosus. Lupus. (2024) 33:1227–34. doi: 10.1177/09612033241272931 39167163

[68] Lisitsyna TA Borisova AB Reshetnyak TM Veltishchev D Diatroptov ME Avdeeva AS . Clinical significance of the determination of antibodies to the NR2 subunit of glutamate N-methyl-D-aspartate receptors (aNMDAR) in patients with systemic lupus erythematosus and antiphospholipid syndrome. Nauchno-Prakt Revmatol. (2025) 63:168–75. doi: 10.47360/1995-4484-2025-168-175

[69] DeGiorgio LA Konstantinov KN Lee SC Hardin JA Volpe BT Diamond B . A subset of lupus anti-DNA antibodies cross-reacts with the NR2 glutamate receptor in systemic lupus erythematosus. Nat Med. (2001) 7:1189–93. doi: 10.1038/nm1101-1189 11689882

[70] Tay SH Fairhurst A-M Mak A . Clinical utility of circulating anti-N-methyl-d-aspartate receptor subunits NR2A/B antibody for the diagnosis of neuropsychiatric syndromes in systemic lupus erythematosus and Sjögren’s syndrome: An updated meta-analysis. Autoimmun Rev. (2017) 16:114–22. doi: 10.1016/j.autrev.2016.12.002 27988431

[71] Arinuma Y Yanagida T Hirohata S . Association of cerebrospinal fluid anti-NR2 glutamate receptor antibodies with diffuse neuropsychiatric systemic lupus erythematosus. Arthritis Rheum. (2008) 58:1130–5. doi: 10.1002/art.23399 18383393

[72] Carroll KR Mizrachi M Simmons S Toz B Kowal C Wingard J . Lupus autoantibodies initiate neuroinflammation sustained by continuous HMGB1:RAGE signaling and reversed by increased LAIR-1 expression. Nat Immunol. (2024) 25:671–81. doi: 10.1038/s41590-024-01772-6 38448779 PMC11141703

[73] Dalmau J Gleichman AJ Hughes EG Rossi JE Peng X Lai M . Anti-NMDA-receptor encephalitis: case series and analysis of the effects of antibodies. Lancet Neurol. (2008) 7:1091–8. doi: 10.1016/S1474-4422(08)70224-2 18851928 PMC2607118

[74] Wang H Xie C Deng B Ding J Li N Kou Z . Structural basis for antibody-mediated NMDA receptor clustering and endocytosis in autoimmune encephalitis. Nat Struct Mol Biol. (2024) 31:1987–96. doi: 10.1038/s41594-024-01387-3 39227720 PMC11638077

[75] Maccioni RB Cambiazo V . Role of microtubule-associated proteins in the control of microtubule assembly. Physiol Rev. (1995) 75:835–64. doi: 10.1152/physrev.1995.75.4.835 7480164

[76] Williams RC Sugiura K Tan EM . Antibodies to microtubule-associated protein 2 in patients with neuropsychiatric systemic lupus erythematosus. Arthritis Rheum. (2004) 50:1239–47. doi: 10.1002/art.20156 15077307

[77] Sasajima T Watanabe H Sato S Sato Y Ohira H . Anti-triosephosphate isomerase antibodies in cerebrospinal fluid are associated with neuropsychiatric lupus. J Neuroimmunol. (2006) 181:150–6. doi: 10.1016/j.jneuroim.2006.09.003 17064784

[78] Watanabe H Seino T Sato Y . Antibodies to triosephosphate isomerase in patients with neuropsychiatric lupus. Biochem Biophys Res Commun. (2004) 321:949–53. doi: 10.1016/j.bbrc.2004.07.051 15358119

[79] Cutillo G Saariaho A-H Meri S . Physiology of gangliosides and the role of antiganglioside antibodies in human diseases. Cell Mol Immunol. (2020) 17:313–22. doi: 10.1038/s41423-020-0388-9 32152553 PMC7109116

[80] Chen Y Wu F Hou L Ma S He X . Antiganglioside antibodies in cerebrospinal fluid of children with neuropsychiatric lupus erythematosus. Chin Med J (Engl). (1997) 110:594–7. 9594260

[81] Engel S Boedecker S Marczynski P Bittner S Steffen F Weinmann A . Association of serum neurofilament light chain levels and neuropsychiatric manifestations in systemic lupus erythematosus. Ther Adv Neurol Disord. (2021) 14:17562864211051497. doi: 10.1177/17562864211051497 34707690 PMC8543555

[82] Balajková V Prokopcová A Elisak M Mojžišová H Pavelka K Olejárová M . Elevated serum neurofilament light chain levels in patients with neuropsychiatric systemic lupus erythematosus: a cross-sectional study. Lupus Sci Med. (2025) 12:e001309. doi: 10.1136/lupus-2024-001309 39832906 PMC11752028

[83] Fanouriakis A Kostopoulou M Anders H-J Andersen J Aringer M Beresford MW . EULAR recommendations for the management of systemic lupus erythematosus with kidney involvement: 2025 update. Ann Rheum Dis. (2026) 85:75–90. doi: 10.1016/j.ard.2025.09.007 41107121

[84] Rovin BH Ayoub IM Chan TM Liu Z-H Mejía-Vilet JM Floege J . KDIGO 2024 clinical practice guideline for the management of LUPUS NEPHRITIS. Kidney Int. (2024) 105:S1–S69. doi: 10.1016/j.kint.2023.09.002 38182286

[85] Vrabie A Obrişcă B Sorohan BM Ismail G . Biomarkers in Lupus Nephritis: An evidence-based comprehensive review. Life (Basel). (2025) 15:1497. doi: 10.3390/life15101497 41157170 PMC12565261

[86] Rekvig OP . Anti-dsDNA antibodies as a classification criterion and a diagnostic marker for systemic lupus erythematosus: critical remarks. Clin Exp Immunol. (2014) 179:5–10. doi: 10.1111/cei.12296 24533624 PMC4260890

[87] Caster DJ Powell DW . Utilization of biomarkers in lupus nephritis. Adv Chronic Kidney Dis. (2019) 26:351–9. doi: 10.1053/j.ackd.2019.09.001 31733719 PMC7001747

[88] Alduraibi FK Tsokos GC . Lupus nephritis biomarkers: A critical review. Int J Mol Sci. (2024) 25:805. doi: 10.3390/ijms25020805 38255879 PMC10815779

[89] Holers VM . Anti-C1q autoantibodies amplify pathogenic complement activation in systemic lupus erythematosus. J Clin Invest. (2004) 114:616–9. doi: 10.1172/JCI22820 15343378 PMC514596

[90] Calatroni M Conte E Stella M De Liso F Reggiani F Moroni G . Clinical and immunological biomarkers can identify proliferative changes and predict renal flares in lupus nephritis. Arthritis Res Ther. (2025) 27:72. doi: 10.1186/s13075-025-03536-5 40165257 PMC11956191

[91] Trouw LA Groeneveld TWL Seelen MA Duijs JMGJ Bajema IM Prins FA . Anti-C1q autoantibodies deposit in glomeruli but are only pathogenic in combination with glomerular C1q-containing immune complexes. J Clin Invest. (2004) 114:679–88. doi: 10.1172/JCI21075 15343386 PMC514584

[92] Eggleton P Ukoumunne OC Cottrell I Khan A Maqsood S Thornes J . Autoantibodies against C1q as a diagnostic measure of lupus nephritis: Systematic review and meta-analysis. J Clin Cell Immunol. (2014) 5:210. doi: 10.4172/2155-9899.1000210 24955287 PMC4062947

[93] Szalai AJ . C-reactive protein (CRP) and autoimmune disease: facts and conjectures. Clin Dev Immunol. (2004) 11:221–6. doi: 10.1080/17402520400001751 15559367 PMC2486333

[94] Yuan M Tan Y Zhao M . The role of anti-mCRP autoantibodies in lupus nephritis. Kidney Dis (Basel). (2023) 9:317–25. doi: 10.1159/000530928 37901707 PMC10601961

[95] Sjöwall C Zickert A Skogh T Wetterö J Gunnarsson I . Serum levels of autoantibodies against C-reactive protein correlate with renal disease activity and response to therapy in lupus nephritis. Arthritis Res Ther. (2009) 11:R188. doi: 10.1186/ar2880 20003354 PMC3003497

[96] Pesickova SS Rysava R Lenicek M Vitek L Potlukova E Hruskova Z . Prognostic value of anti-CRP antibodies in lupus nephritis in long-term follow-up. Arthritis Res Ther. (2015) 17:371. doi: 10.1186/s13075-015-0879-8 26704903 PMC4718018

[97] Liu X-L Tan Y Yu F Ji S-R Zhao M-H . Combination of anti-C1qA08 and anti-mCRP a.a.35-47 antibodies is associated with renal prognosis of patients with lupus nephritis. Front Immunol. (2023) 14:1181561. doi: 10.3389/fimmu.2023.1181561 37138875 PMC10150958

[98] Rai K Khatri R Jose A Konkar H Nadkar M Rajadhyaksha A . Lectin pathway components and autoantibodies as novel immunological biomarkers in systemic lupus erythematosus (SLE) patients from Western India. Sci Rep. (2025) 15:37940. doi: 10.1038/s41598-025-10891-5 41168241 PMC12575780

[99] Colliard S Jourde-Chiche N Clavarino G Sarrot-Reynauld F Gout E Deroux A . Autoantibodies targeting Ficolin-2 in systemic lupus erythematosus patients with active nephritis. Arthritis Care Res (Hoboken). (2018) 70:1263–8. doi: 10.1002/acr.23449 29045037

[100] Plawecki M Lheritier E Clavarino G Jourde-Chiche N Ouili S Paul S . Association between the presence of autoantibodies targeting Ficolin-3 and active nephritis in patients with systemic lupus erythematosus. PLoS One. (2016) 11:e0160879. doi: 10.1371/journal.pone.0160879 27631981 PMC5025237

[101] Elkoumi MA Emam AA Allah MAN Sherif AH Abdelaal NM Mosabah A . Association of ficolin-2 gene polymorphisms and susceptibility to systemic lupus erythematosus in Egyptian children and adolescents: a multicenter study. Lupus. (2019) 28:995–1002. doi: 10.1177/0961203319856089 31184250

[102] Deocharan B Qing X Lichauco J Putterman C . Alpha-actinin is a cross-reactive renal target for pathogenic anti-DNA antibodies. J Immunol. (2002) 168:3072–8. doi: 10.4049/jimmunol.168.6.3072 11884481

[103] Zhao Z Weinstein E Tuzova M Davidson A Mundel P Marambio P . Cross-reactivity of human lupus anti-DNA antibodies with alpha-actinin and nephritogenic potential. Arthritis Rheum. (2005) 52:522–30. doi: 10.1002/art.20862 15693007

[104] Manson JJ Ma A Rogers P Mason LJ Berden JH van der Vlag J . Relationship between anti-dsDNA, anti-nucleosome and anti-alpha-actinin antibodies and markers of renal disease in patients with lupus nephritis: a prospective longitudinal study. Arthritis Res Ther. (2009) 11:R154. doi: 10.1186/ar2831 19828047 PMC2787270

[105] Seret G Cañas F Pougnet-Di Costanzo L Hanrotel-Saliou C Jousse-Joulin S Le Meur Y . Anti-alpha-actinin antibodies are part of the anti-cell membrane antibody spectrum that characterize patients with lupus nephritis. J Autoimmun. (2015) 61:54–61. doi: 10.1016/j.jaut.2015.05.009 26071203

[106] Krishnan MR Wang C Marion TN . Anti-DNA autoantibodies initiate experimental lupus nephritis by binding directly to the glomerular basement membrane in mice. Kidney Int. (2012) 82:184–92. doi: 10.1038/ki.2011.484 22297676 PMC3343188

[107] Kim H-J Hong Y-H Kim Y-J Kim H-S Park J-W Do J-Y . Anti-heparan sulfate antibody and functional loss of glomerular heparan sulfate proteoglycans in lupus nephritis. Lupus. (2017) 26:815–24. doi: 10.1177/0961203316678674 28420046

[108] Bruschi M Sinico RA Moroni G Pratesi F Migliorini P Galetti M . Glomerular autoimmune multicomponents of human lupus nephritis *in vivo*: α-enolase and annexin AI. J Am Soc Nephrol. (2014) 25:2483–98. doi: 10.1681/ASN.2013090987 24790181 PMC4214525

[109] Bruschi M Galetti M Sinico RA Moroni G Bonanni A Radice A . Glomerular autoimmune multicomponents of human lupus nephritis *in vivo* (2): planted antigens. J Am Soc Nephrol. (2015) 26:1905–24. doi: 10.1681/ASN.2014050493 25398787 PMC4520170

[110] Gomes MF Mardones C Xipell M Blasco M Solé M Espinosa G . The extent of tubulointerstitial inflammation is an independent predictor of renal survival in lupus nephritis. J Nephrol. (2021) 34:1897–905. doi: 10.1007/s40620-021-01007-z 33721269

[111] Kinloch AJ Chang A Ko K Henry Dunand CJ Henderson S Maienschein-Cline M . Vimentin is a dominant target of in situ humoral immunity in human lupus tubulointerstitial nephritis. Arthritis Rheumatol. (2014) 66:3359–70. doi: 10.1002/art.38888 25306868 PMC4264660

[112] Tsai C-Y Wu T-H Ou S-M Lee H-T Shen C-Y Lu C-H . The pathogenesis, potential biomarkers and novel therapeutic strategies for tubulointerstitial nephritis in systemic lupus erythematosus-a narrative review. Int J Mol Sci. (2025) 26:10903. doi: 10.3390/ijms262210903 41303387 PMC12652633

[113] Kinloch AJ Asano Y Mohsin A Henry C Abraham R Chang A . Machine learning to quantify in situ humoral selection in human lupus tubulointerstitial inflammation. Front Immunol. (2020) 11:593177. doi: 10.3389/fimmu.2020.593177 33329582 PMC7731665

[114] Beyze A Larroque C Le Quintrec M . The role of antibody glycosylation in autoimmune and alloimmune kidney diseases. Nat Rev Nephrol. (2024) 20:672–89. doi: 10.1038/s41581-024-00850-0 38961307

[115] Elmgren J Nyberg F . Clinical aspects of cutaneous lupus erythematosus. Front Med (Lausanne). (2022) 9:984229. doi: 10.3389/fmed.2022.984229 36698816 PMC9868707

[116] Shi Z-R Tan G-Z Meng Z Yu M Li K-W Yin J . Association of anti-acidic ribosomal protein P0 and anti-galectin 3 antibodies with the development of skin lesions in systemic lupus erythematosus. Arthritis Rheumatol. (2015) 67:193–203. doi: 10.1002/art.38891 25307291

[117] Contestable JJ Edhegard KD Meyerle JH . Bullous systemic lupus erythematosus: a review and update to diagnosis and treatment. Am J Clin Dermatol. (2014) 15:517–24. doi: 10.1007/s40257-014-0098-0 25358414

[118] Mahla RS Jones EL Dustin LB . Ro60-roles in RNA processing, inflammation, and rheumatic autoimmune diseases. Int J Mol Sci. (2024) 25:7705. doi: 10.3390/ijms25147705 39062948 PMC11277228

[119] Jones EL Laidlaw SM Dustin LB . TRIM21/Ro52 - roles in innate immunity and autoimmune disease. Front Immunol. (2021) 12:738473. doi: 10.3389/fimmu.2021.738473 34552597 PMC8450407

[120] Hung T Pratt GA Sundararaman B Townsend MJ Chaivorapol C Bhangale T . The Ro60 autoantigen binds endogenous retroelements and regulates inflammatory gene expression. Science. (2015) 350:455–9. doi: 10.1126/science.aac7442 26382853 PMC4691329

[121] Gryka-Marton M Szukiewicz D Teliga-Czajkowska J Olesinska M . An overview of neonatal lupus with anti-Ro characteristics. Int J Mol Sci. (2021) 22:9281. doi: 10.3390/ijms22179281 34502221 PMC8431034

[122] Provost TT Watson R Simmons-O’Brien E . Significance of the anti-Ro (SS-A) antibody in evaluation of patients with cutaneous manifestations of a connective tissue disease. J Am Acad Dermatol. (1996) 35:147–69. doi: 10.1016/s0190-9622(96)90315-1 8708014

[123] Cioni P Chretien P Goulvestre C Ballot E Khelifi-Touhami D Mariette X . Isolated anti-SS-B (La) antibodies: rare occurrence and lack of diagnostic value. RMD Open. (2025) 11:e005212. doi: 10.1136/rmdopen-2024-005212 39904568 PMC11795515

[124] Rao L Liu G Li C Li Y Wang Z Zhou Z . Specificity of anti-SSB as a diagnostic marker for the classification of systemic lupus erythematosus. Exp Ther Med. (2013) 5:1710–4. doi: 10.3892/etm.2013.1051 23837059 PMC3702711

[125] Clancy RM Neufing PJ Zheng P O’Mahony M Nimmerjahn F Gordon TP . Impaired clearance of apoptotic cardiocytes is linked to anti-SSA/Ro and -SSB/La antibodies in the pathogenesis of congenital heart block. J Clin Invest. (2006) 116:2413–22. doi: 10.1172/JCI27803 16906225 PMC1533875

[126] Jones SK . Ultraviolet radiation (UVR) induces cell-surface Ro/SSA antigen expression by human keratinocytes *in vitro*: a possible mechanism for the UVR induction of cutaneous lupus lesions. Br J Dermatol. (1992) 126:546–53. doi: 10.1111/j.1365-2133.1992.tb00098.x 1610705

[127] Lee LA Gaither KK Coulter SN Norris DA Harley JB . Pattern of cutaneous immunoglobulin G deposition in subacute cutaneous lupus erythematosus is reproduced by infusing purified anti-Ro (SSA) autoantibodies into human skin-grafted mice. J Clin Invest. (1989) 83:1556–62. doi: 10.1172/JCI114052 2651477 PMC303861

[128] Clancy RM Alvarez D Komissarova E Barrat FJ Swartz J Buyon JP . Ro60-associated single-stranded RNA links inflammation with fetal cardiac fibrosis via ligation of TLRs: a novel pathway to autoimmune-associated heart block. J Immunol. (2010) 184:2148–55. doi: 10.4049/jimmunol.0902248 20089705 PMC3551297

[129] Shi Z Han Y Yin J Zhang Y Jiang Z Zheng L . The diagnostic benefit of antibodies against ribosomal proteins in systemic lupus erythematosus. Adv Rheumatol. (2020) 60:45. doi: 10.1186/s42358-020-00148-2 32859277

[130] Argüeso P Panjwani N . Focus on molecules: galectin-3. Exp Eye Res. (2011) 92:2–3. doi: 10.1016/j.exer.2010.11.009 21111733 PMC3034303

[131] McLeod K Walker JT Hamilton DW . Galectin-3 regulation of wound healing and fibrotic processes: insights for chronic skin wound therapeutics. J Cell Commun Signal. (2018) 12:281–7. doi: 10.1007/s12079-018-0453-7 29372416 PMC5842207

[132] Henderson NC Sethi T . The regulation of inflammation by galectin-3. Immunol Rev. (2009) 230:160–71. doi: 10.1111/j.1600-065X.2009.00794.x 19594635

[133] Liu F-T Yang R-Y Hsu DK . Galectins in acute and chronic inflammation. Ann N Y Acad Sci. (2012) 1253:80–91. doi: 10.1111/j.1749-6632.2011.06386.x 22329844

[134] Yang RY Hsu DK Liu FT . Expression of galectin-3 modulates T-cell growth and apoptosis. Proc Natl Acad Sci USA. (1996) 93:6737–42. doi: 10.1073/pnas.93.13.6737 8692888 PMC39096

[135] Shi Z Zhang Y-P Hong D Qiu X Zheng L Bian L . Anti-galectin-3 antibodies induce skin vascular inflammation via promoting local production of IL-1β in systemic lupus erythematosus. Int Immunopharmacol. (2022) 112:109197. doi: 10.1016/j.intimp.2022.109197 36058031

[136] Gammon WR Murrell DF Jenison MW Padilla KM Prisayanh PS Jones DA . Autoantibodies to type VII collagen recognize epitopes in a fibronectin-like region of the noncollagenous (NC1) domain. J Invest Dermatol. (1993) 100:618–22. doi: 10.1111/1523-1747.ep12472291 7684054

[137] Grabell DA Matthews LA Yancey KB Chong BF . Detection of type VII collagen autoantibodies before the onset of bullous systemic lupus erythematosus. JAMA Dermatol. (2015) 151:539–43. doi: 10.1001/jamadermatol.2014.4409 25671758 PMC4874328

[138] Chan LS Lapiere JC Chen M Traczyk T Mancini AJ Paller AS . Bullous systemic lupus erythematosus with autoantibodies recognizing multiple skin basement membrane components, bullous pemphigoid antigen 1, laminin-5, laminin-6, and type VII collagen. Arch Dermatol. (1999) 135:569–73. doi: 10.1001/archderm.135.5.569 10328198

[139] Ambler WG Kaplan MJ . Vascular damage in systemic lupus erythematosus. Nat Rev Nephrol. (2024) 20:251–65. doi: 10.1038/s41581-023-00797-8 38172627 PMC11391830

[140] Baumgarth N . The double life of a B-1 cell: self-reactivity selects for protective effector functions. Nat Rev Immunol. (2011) 11:34–46. doi: 10.1038/nri2901 21151033

[141] Enghard P Humrich JY Chu VT Grussie E Hiepe F Burmester G-R . Class switching and consecutive loss of dsDNA-reactive B1a B cells from the peritoneal cavity during murine lupus development. Eur J Immunol. (2010) 40:1809–18. doi: 10.1002/eji.200940050 20333624

[142] Anania C Gustafsson T Hua X Su J Vikström M de Faire U . Increased prevalence of vulnerable atherosclerotic plaques and low levels of natural IgM antibodies against phosphorylcholine in patients with systemic lupus erythematosus. Arthritis Res Ther. (2010) 12:R214. doi: 10.1186/ar3193 21092251 PMC3046524

[143] Svenungsson E Engelbertsen D Wigren M Gustafsson JT Gunnarsson I Elvin K . Decreased levels of autoantibodies against apolipoprotein B-100 antigens are associated with cardiovascular disease in systemic lupus erythematosus. Clin Exp Immunol. (2015) 181:417–26. doi: 10.1111/cei.12651 25959453 PMC4557377

[144] Wirestam L Jönsson F Enocsson H Svensson C Weiner M Wetterö J . Limited association between antibodies to oxidized low-density lipoprotein and vascular affection in patients with established systemic lupus erythematosus. Int J Mol Sci. (2023) 24:8987. doi: 10.3390/ijms24108987 37240332 PMC10219253

[145] de Carvalho JF Borba EF Viana VST Bueno C Leon EP Bonfá E . Anti-lipoprotein lipase antibodies: a new player in the complex atherosclerotic process in systemic lupus erythematosus? Arthritis Rheum. (2004) 50:3610–5. doi: 10.1002/art.20630 15529371

[146] van der Zee JM Siegert CE de Vreede TA Daha MR Breedveld FC . Characterization of anti-endothelial cell antibodies in systemic lupus erythematosus (SLE). Clin Exp Immunol. (1991) 84:238–44. doi: 10.1111/j.1365-2249.1991.tb08155.x 2025951 PMC1535404

[147] Song J Park YB Lee WK Lee KH Lee SK . Clinical associations of anti-endothelial cell antibodies in patients with systemic lupus erythematosus. Rheumatol Int. (2000) 20:1–7. doi: 10.1007/s002960000060 11149654

[148] Cines DB Lyss AP Reeber M Bina M DeHoratius RJ . Presence of complement-fixing anti-endothelial cell antibodies in systemic lupus erythematosus. J Clin Invest. (1984) 73:611–25. doi: 10.1172/JCI111251 6368583 PMC425060

[149] Praprotnik S Blank M Meroni PL Rozman B Eldor A Shoenfeld Y . Classification of anti-endothelial cell antibodies into antibodies against microvascular and macrovascular endothelial cells: the pathogenic and diagnostic implications. Arthritis Rheum. (2001) 44:1484–94. doi: 10.1002/1529-0131(200107)44:7&lt;1484::AID-ART269>3.0.CO;2-Q 11465698

[150] Bouillet L Baudet AE Deroux A Sidibé A Dumestre-Perard C Mannic T . Auto-antibodies to vascular endothelial cadherin in humans: association with autoimmune diseases. Lab Invest. (2013) 93:1194–202. doi: 10.1038/labinvest.2013.106 24061286

[151] Guo L Li M Chen Y Wang Q Tian Z Pan S . Anti-endothelin receptor type A autoantibodies in systemic lupus erythematosus-associated pulmonary arterial hypertension. Arthritis Rheumatol. (2015) 67:2394–402. doi: 10.1002/art.39212 26018988

[152] Carmona-Rivera C Zhao W Yalavarthi S Kaplan MJ . Neutrophil extracellular traps induce endothelial dysfunction in systemic lupus erythematosus through the activation of matrix metalloproteinase-2. Ann Rheum Dis. (2015) 74:1417–24. doi: 10.1136/annrheumdis-2013-204837 24570026 PMC4143484

[153] de Laat B Derksen RHWM van Lummel M Pennings MTT de Groot PG . Pathogenic anti-beta2-glycoprotein I antibodies recognize domain I of beta2-glycoprotein I only after a conformational change. Blood. (2006) 107:1916–24. doi: 10.1182/blood-2005-05-1943 16269621

[154] Atsumi T Ieko M Bertolaccini ML Ichikawa K Tsutsumi A Matsuura E . Association of autoantibodies against the phosphatidylserine-prothrombin complex with manifestations of the antiphospholipid syndrome and with the presence of lupus anticoagulant. Arthritis Rheum. (2000) 43:1982–93. doi: 10.1002/1529-0131(200009)43:9<1982::AID-ANR9>3.0.CO;2-2 11014348

[155] Pham M Orsolini G Crowson C Snyder M Pruthi R Moder K . Anti-phosphatidylserine prothrombin antibodies as a predictor of the lupus anticoagulant in an all-comer population. J Thromb Haemost. (2022) 20:2070–4. doi: 10.1111/jth.15792 35722911

[156] Zhang Y Su Y Guo H Wang L Li A Wang C . Anti-phosphatidylserine/prothrombin complex antibodies (aPS/PT) increase the risk for thrombosis based on lupus anticoagulant positivity. Clin Biochem. (2023) 112:17–23. doi: 10.1016/j.clinbiochem.2022.12.007 36535385

[157] Cesarman-Maus G Ríos-Luna NP Deora AB Huang B Villa R Cravioto MC . Autoantibodies against the fibrinolytic receptor, annexin 2, in antiphospholipid syndrome. Blood. (2006) 107:4375–82. doi: 10.1182/blood-2005-07-2636 16493010 PMC1895790

[158] Ao W Zheng H Chen X-W Shen Y Yang C-D . Anti-annexin II antibody is associated with thrombosis and/or pregnancy morbidity in antiphospholipid syndrome and systemic lupus erythematosus with thrombosis. Rheumatol Int. (2011) 31:865–9. doi: 10.1007/s00296-010-1379-4 20229030

[159] Rand JH Wu X-X Quinn AS Ashton AW Chen PP Hathcock JJ . Hydroxychloroquine protects the annexin A5 anticoagulant shield from disruption by antiphospholipid antibodies: evidence for a novel effect for an old antimalarial drug. Blood. (2010) 115:2292–9. doi: 10.1182/blood-2009-04-213520 19965621 PMC2844013

[160] Cederholm A Frostegård J . Annexin A5 in cardiovascular disease and systemic lupus erythematosus. Immunobiology. (2005) 210:761–8. doi: 10.1016/j.imbio.2005.10.007 16325495

[161] Hrycek A Cieślik P . Annexin A5 and anti-annexin antibodies in patients with systemic lupus erythematosus. Rheumatol Int. (2012) 32:1335–42. doi: 10.1007/s00296-011-1793-2 21298269

[162] Amarnani R Yeoh S-A Denneny EK Wincup C . Lupus and the lungs: The assessment and management of pulmonary manifestations of systemic lupus erythematosus. Front Med (Lausanne). (2020) 7:610257. doi: 10.3389/fmed.2020.610257 33537331 PMC7847931

[163] Mormile I Nazzaro G Filippelli M Della Casa F Mormile M de Paulis A . Pulmonary involvement in systemic lupus erythematosus: A potentially overlooked condition. Biomedicines. (2025) 13:1485. doi: 10.3390/biomedicines13061485 40564204 PMC12191131

[164] Depascale R Del Frate G Gasparotto M Manfrè V Gatto M Iaccarino L . Diagnosis and management of lung involvement in systemic lupus erythematosus and Sjögren’s syndrome: a literature review. Ther Adv Musculoskelet Dis. (2021) 13:1759720X211040696. doi: 10.1177/1759720X211040696 34616495 PMC8488521

[165] Hannah JR D’Cruz DP . Pulmonary complications of systemic lupus erythematosus. Semin Respir Crit Care Med. (2019) 40:227–34. doi: 10.1055/s-0039-1685537 31137062

[166] Good JT King TE Antony VB Sahn SA . Lupus pleuritis. Clinical features and pleural fluid characteristics with special reference to pleural fluid antinuclear antibodies. Chest. (1983) 84:714–8. doi: 10.1378/chest.84.6.714 6605838

[167] Chen D-Y Huang Y-H Chen Y-M Chen JJW Yang T-Y Chang G-C . ANA positivity and complement level in pleural fluid are potential diagnostic markers in discriminating lupus pleuritis from pleural effusion of other aetiologies. Lupus Sci Med. (2021) 8:e000562. doi: 10.1136/lupus-2021-000562 34785570 PMC8596033

[168] Tselios K Urowitz MB . Cardiovascular and pulmonary manifestations of systemic lupus erythematosus. Curr Rheumatol Rev. (2017) 13:206–18. doi: 10.2174/1573397113666170704102444 28675998

[169] Zamora MR Warner ML Tuder R Schwarz MI . Diffuse alveolar hemorrhage and systemic lupus erythematosus. Clinical presentation, histology, survival, and outcome. Medicine (Baltimore). (1997) 76:192–202. doi: 10.1097/00005792-199705000-00005 9193454

[170] Andrade C Mendonça T Farinha F Correia J Marinho A Almeida I . Alveolar hemorrhage in systemic lupus erythematosus: a cohort review. Lupus. (2016) 25:75–80. doi: 10.1177/0961203315605365 26385219

[171] Jiang M Chen R Zhao L Zhang X . Risk factors for mortality of diffuse alveolar hemorrhage in systemic lupus erythematosus: a systematic review and meta-analysis. Arthritis Res Ther. (2021) 23:57. doi: 10.1186/s13075-021-02435-9 33593433 PMC7885396

[172] González-Treviño M Figueroa-Parra G Yang JX Prokop LJ Gamal SM García MA . Association between antiphospholipid antibodies and diffuse alveolar haemorrhage risk in systemic lupus erythematosus: a systematic review and meta-analysis. Rheumatology (Oxford). (2025) 64:1598–608. doi: 10.1093/rheumatology/keae632 39558618 PMC11962882

[173] Nayebirad S Mohamadi A Yousefi-Koma H Javadi M Farahmand K Atef-Yekta R . Association of anti-Ro52 autoantibody with interstitial lung disease in autoimmune diseases: a systematic review and meta-analysis. BMJ Open Respir Res. (2023) 10:e002076. doi: 10.1136/bmjresp-2023-002076 38030264 PMC10689422

[174] Deneuville L Mageau A Debray MP Sacre K Costedoat-Chalumeau N Hachulla E . Chronic interstitial lung disease associated with systemic lupus erythematosus: A multicentric study of 89 cases. Respirology. (2024) 29:713–21. doi: 10.1111/resp.14703 38494831

[175] Runyon BA LaBrecque DR Anuras S . The spectrum of liver disease in systemic lupus erythematosus. Report of 33 histologically-proved cases and review of the literature. Am J Med. (1980) 69:187–94. doi: 10.1016/0002-9343(80)90378-2 7405944

[176] Bessone F Poles N Roma MG . Challenge of liver disease in systemic lupus erythematosus: Clues for diagnosis and hints for pathogenesis. World J Hepatol. (2014) 6:394–409. doi: 10.4254/wjh.v6.i6.394 25018850 PMC4081614

[177] Imran S Thabah MM Azharudeen M Ramesh A Bobby Z Negi VS . Liver abnormalities in systemic lupus erythematosus: A prospective observational study. Cureus. (2021) 13:e15691. doi: 10.7759/cureus.15691 34277279 PMC8284411

[178] Adiga A Nugent K . Lupus hepatitis and autoimmune hepatitis (Lupoid hepatitis). Am J Med Sci. (2017) 353:329–35. doi: 10.1016/j.amjms.2016.10.014 28317620

[179] Arnett FC Reichlin M . Lupus hepatitis: an under-recognized disease feature associated with autoantibodies to ribosomal P. Am J Med. (1995) 99:465–72. doi: 10.1016/s0002-9343(99)80221-6 7485202

[180] Koren E Reichlin MW Koscec M Fugate RD Reichlin M . Autoantibodies to the ribosomal P proteins react with a plasma membrane-related target on human cells. J Clin Invest. (1992) 89:1236–41. doi: 10.1172/JCI115707 1313450 PMC442983

[181] Koscec M Koren E Wolfson-Reichlin M Fugate RD Trieu E Targoff IN . Autoantibodies to ribosomal P proteins penetrate into live hepatocytes and cause cellular dysfunction in culture. J Immunol. (1997) 159:2033–41. doi: 10.4049/jimmunol.159.4.2033 9257871

[182] Kushimoto K Nagasawa K Ueda A Mayumi T Ishii Y Yamauchi Y . Liver abnormalities and liver membrane autoantibodies in systemic lupus erythematosus. Ann Rheum Dis. (1989) 48:946–52. doi: 10.1136/ard.48.11.946 2596885 PMC1003919

[183] Ahmad A Heijke R Eriksson P Wirestam L Kechagias S Dahle C . Autoantibodies associated with primary biliary cholangitis are common among patients with systemic lupus erythematosus even in the absence of elevated liver enzymes. Clin Exp Immunol. (2021) 203:22–31. doi: 10.1111/cei.13512 32910463 PMC7744498

[184] Terjung B Spengler U . Atypical p-ANCA in PSC and AIH: a hint toward a “leaky gut”? Clin Rev Allergy Immunol. (2009) 36:40–51. doi: 10.1007/s12016-008-8088-8 18626795

[185] Liu Y Yu J Oaks Z Marchena-Mendez I Francis L Bonilla E . Liver injury correlates with biomarkers of autoimmunity and disease activity and represents an organ system involvement in patients with systemic lupus erythematosus. Clin Immunol. (2015) 160:319–27. doi: 10.1016/j.clim.2015.07.001 26160213 PMC4583603

[186] Buckner JH . Antigen-specific immunotherapies for autoimmune disease. Nat Rev Rheumatol. (2025) 21:88–97. doi: 10.1038/s41584-024-01201-w 39681709

[187] Mackensen A Müller F Mougiakakos D Böltz S Wilhelm A Aigner M . Anti-CD19 CAR T cell therapy for refractory systemic lupus erythematosus. Nat Med. (2022) 28:2124–32. doi: 10.1038/s41591-022-02017-5 36109639

[188] Liu H Zhang H Xu Y Wang J Zhang W Lei B . Application of BCMA CAR-T in systemic lupus erythematosus. Clin Rheumatol. (2026) 45:3289–94. doi: 10.1007/s10067-026-08121-y 42010209

[189] Wang W He S Zhang W Zhang H DeStefano VM Wada M . BCMA-CD19 compound CAR T cells for systemic lupus erythematosus: a phase 1 open-label clinical trial. Ann Rheum Dis. (2024) 83:1304–14. doi: 10.1136/ard-2024-225785 38777376

[190] Feng J Huo D Hong R Jin X Cao H Shao M . Co-infusion of CD19-targeting and BCMA-targeting CAR-T cells for treatment-refractory systemic lupus erythematosus: a phase 1 trial. Nat Med. (2025) 31:3725–36. doi: 10.1038/s41591-025-03937-8 40993243

[191] Stepanov AV Kalinin RS Shipunova VO Zhang D Xie J Rubtsov YP . Switchable targeting of solid tumors by BsCAR T cells. Proc Natl Acad Sci USA. (2022) 119:e2210562119. doi: 10.1073/pnas.2210562119 36343224 PMC9674235

[192] Wang Q Xiao ZX Zheng X Wang G Yang L Shi L . *In vivo* CD19 CAR T-cell therapy for refractory systemic lupus erythematosus. N Engl J Med. (2025) 393:1542–4. doi: 10.1056/NEJMc2509522 40961420

[193] Chung JB Brudno JN Borie D Kochenderfer JN . Chimeric antigen receptor T cell therapy for autoimmune disease. Nat Rev Immunol. (2024) 24:830–45. doi: 10.1038/s41577-024-01035-3 38831163 PMC12176013

[194] Bloom O Cheng KF He M Papatheodorou A Volpe BT Diamond B . Generation of a unique small molecule peptidomimetic that neutralizes lupus autoantibody activity. Proc Natl Acad Sci USA. (2011) 108:10255–9. doi: 10.1073/pnas.1103555108 21646518 PMC3121823

[195] Wang H Lu M Zhai S Wu K Peng L Yang J . ALW peptide ameliorates lupus nephritis in MRL/lpr mice. Arthritis Res Ther. (2019) 21:261. doi: 10.1186/s13075-019-2038-0 31791413 PMC6889545

[196] Urowitz MB Isenberg DA Wallace DJ . Safety and efficacy of hCDR1 (Edratide) in patients with active systemic lupus erythematosus: results of phase II study. Lupus Sci Med. (2015) 2:e000104. doi: 10.1136/lupus-2015-000104 26301100 PMC4538379

[197] Zimmer R Scherbarth HR Rillo OL Gomez-Reino JJ Muller S . Lupuzor/P140 peptide in patients with systemic lupus erythematosus: a randomised, double-blind, placebo-controlled phase IIb clinical trial. Ann Rheum Dis. (2013) 72:1830–5. doi: 10.1136/annrheumdis-2012-202460 23172751 PMC3812851

[198] Kang H-K Liu M Datta SK . Low-dose peptide tolerance therapy of lupus generates plasmacytoid dendritic cells that cause expansion of autoantigen-specific regulatory T cells and contraction of inflammatory Th17 cells. J Immunol. (2007) 178:7849–58. doi: 10.4049/jimmunol.178.12.7849 17548623

[199] Lim Z Ting Y Vincent F Rischmueller M Morand E Ooi J . Genetically-engineered Ro-specific regulatory T cells to treat primary Sjögren’s disease [abstract]. Arthritis Rheumatol. (2024) 76(Suppl 9). Available online at: https://acrabstracts.org/abstract/genetically-engineered-ro-specific-regulatory-t-cells-to-treat-primary-sjogrens-disease/ (Accessed June 29, 2026).

[200] Sole C Royo M Sandoval S Moline T Gabaldon A Cortes-Hernandez J . Precise targeting of autoantigen-specific B cells in lupus nephritis with chimeric autoantibody receptor T cells. Int J Mol Sci. (2024) 25:4226. doi: 10.3390/ijms25084226 38673811 PMC11050013

[201] Meng H Sun X Song Y Zou J An G Jin Z . La/SSB chimeric autoantibody receptor modified NK92MI cells for targeted therapy of autoimmune disease. Clin Immunol. (2018) 192:40–9. doi: 10.1016/j.clim.2018.04.006 29673902

[202] Perico L Casiraghi F Sônego F Todeschini M Corna D Cerullo D . Bi-specific autoantigen-T cell engagers as targeted immunotherapy for autoreactive B cell depletion in autoimmune diseases. Front Immunol. (2024) 15:1335998. doi: 10.3389/fimmu.2024.1335998 38469301 PMC10926275

[203] Xia Y Liu J Pearlman A Mog B Shaw E Kaeo K . Bispecific Autoantigen-T Cell Engagers (BaiTE) to selectively target autoreactive B cells in antiphospholipid syndrome [abstract]. Arthritis Rheumatol. (2024) 76(Suppl 9). Available online at: https://acrabstracts.org/abstract/bispecific-autoantigen-t-cell-engagers-baite-to-selectively-target-autoreactive-b-cells-in-antiphospholipid-syndrome/ (Accessed June 29, 2026).

[204] Mog B Shaw E Hwang M Pearlman A DiNapoli S Paul S . Chimeric Autoantigen-T Cell Receptor (CATCR)-T cell therapies to selectively target autoreactive B cells [abstract]. Arthritis Rheumatol. (2022) 76(Suppl 9). Available online at: https://acrabstracts.org/abstract/chimeric-autoantigen-t-cell-receptor-catcr-t-cell-therapies-to-selectively-target-autoreactive-b-cells/ (Accessed June 29, 2026).

[205] Liu J Xia Y Mog BJ Gliech C Shaw E Ferris D . Precision targeting of autoreactive B cells in systemic lupus erythematosus using anti-9G4 idiotope synthetic immune receptor T cells. BioRxiv. (2025) 2025.10.19.682634. doi: 10.1101/2025.10.19.682634 41279526 PMC12632787

[206] Liu J Xia Y Ferris D Shaw E Mog B Pearlman A . T cell-engaging bispecific antibodies to target autoreactive 9G4 idiotope B cells in systemic lupus erythematosus [abstract]. Arthritis Rheumatol. (2024) 76(Suppl 9). Available online at: https://acrabstracts.org/abstract/t-cell-engaging-bispecific-antibodies-to-target-autoreactive-9g4-idiotope-b-cells-in-systemic-lupus-erythematosus/ (Accessed June 29, 2026).

[207] Fasano S Milone A Nicoletti GF Isenberg DA Ciccia F . Precision medicine in systemic lupus erythematosus. Nat Rev Rheumatol. (2023) 19:331–42. doi: 10.1038/s41584-023-00948-y 37041269

[208] Giannouli S Voulgarelis M Ziakas PD Tzioufas AG . Anaemia in systemic lupus erythematosus: from pathophysiology to clinical assessment. Ann Rheum Dis. (2006) 65:144–8. doi: 10.1136/ard.2005.041673 16079164 PMC1798007

[209] Li J Peng L Wu L Ding Y Duan X Xu J . Antiphospholipid antibodies as potential predictors of disease severity and poor prognosis in systemic lupus erythematosus-associated thrombocytopenia: results from a real-world CSTAR cohort study. Arthritis Res Ther. (2024) 26:67. doi: 10.1186/s13075-024-03305-w 38475924 PMC10929102

[210] Ziakas PD Giannouli S Zintzaras E Tzioufas AG Voulgarelis M . Lupus thrombocytopenia: clinical implications and prognostic significance. Ann Rheum Dis. (2005) 64:1366–9. doi: 10.1136/ard.2004.033100 16100344 PMC1755663

[211] Kokori SIG Ioannidis JPA Voulgarelis M Tzioufas AG Moutsopoulos HM . Autoimmune hemolytic anemia in patients with systemic lupus erythematosus. Am J Med. (2000) 108:198–204. doi: 10.1016/S0002-9343(99)00413-1 10723973

[212] Kandane-Rathnayake R Zolio L Choi JI Golder V Louthrenoo W Chen Y-H . Predictors of damage accrual by organ domain in systemic lupus erythematosus. Rheumatology (Oxford). (2026) 65:keaf610. doi: 10.1093/rheumatology/keaf610 41252474

[213] Katechis S Nikolopoulos D Flouda S Adamichou C Chavatza K Kapsala N . Can we predict kidney involvement in patients with systemic lupus erythematosus? A retrospective cohort study with independent validation. Rheumatology (Oxford). (2025) 64:2715–22. doi: 10.1093/rheumatology/keae278 38745434

[214] Garrido-Mesa J Brown MA . Antigen-driven T cell responses in rheumatic diseases: insights from T cell receptor repertoire studies. Nat Rev Rheumatol. (2025) 21:157–73. doi: 10.1038/s41584-025-01218-9 39920282

[215] Arshad S Cameron B Joglekar AV . Immunopeptidomics for autoimmunity: unlocking the chamber of immune secrets. NPJ Syst Biol Appl. (2025) 11:10. doi: 10.1038/s41540-024-00482-x 39833247 PMC11747513

[216] Wu C Jiang S Chen Z Li T Gu X Dai M . Single-cell transcriptomics reveal potent extrafollicular B cell response linked with granzyme K+ CD8 T cell activation in lupus kidney. Ann Rheum Dis. (2025) 84:451–66. doi: 10.1136/ard-2024-225876 39419536

[217] Zheng M Hu Z Zhou W Kong Y Wu R Zhang B . Single-cell transcriptome reveals immunopathological cell composition of skin lesions in subacute cutaneous lupus erythematosus. Clin Immunol. (2022) 245:109172. doi: 10.1016/j.clim.2022.109172 36343898

[218] Liu Q Wu L Hao S Deng Y Liu X Shang S . Single-cell profiling unveils nephritis-related circulating immunological signatures in systemic lupus erythematosus patients. Commun Biol. (2026) 9:155. doi: 10.1038/s42003-025-09431-8 41490945 PMC12868680

[219] Vinuesa CG Shen N Ware T . Genetics of SLE: mechanistic insights from monogenic disease and disease-associated variants. Nat Rev Nephrol. (2023) 19:558–72. doi: 10.1038/s41581-023-00732-x 37438615

[220] Stensballe A . Toward precision health in autoimmunity and immune-related adverse events: The autoantibody reactome, spatial omics, and multimodal data integration. Biomedicines. (2026) 14:1129. doi: 10.3390/biomedicines14051129 42193454 PMC13204632

